# The impact of tonic GABA_A_ receptor-mediated inhibition on neuronal excitability varies across brain region and cell type

**DOI:** 10.3389/fncir.2014.00003

**Published:** 2014-02-03

**Authors:** Vallent Lee, Jamie Maguire

**Affiliations:** ^1^Medical Scientist Training Program and Graduate Program in Neuroscience, Sackler School of Graduate Biomedical Sciences, Tufts UniversityBoston, MA, USA; ^2^Department of Neuroscience, Tufts University School of MedicineBoston, MA, USA

**Keywords:** tonic inhibition, GABA, neurosteroids, neuronal excitability, epilepsy

## Abstract

The diversity of GABA_A_ receptor (GABA_A_R) subunits and the numerous configurations during subunit assembly give rise to a variety of receptors with different functional properties. This heterogeneity results in variations in GABAergic conductances across numerous brain regions and cell types. Phasic inhibition is mediated by synaptically-localized receptors with a low affinity for GABA and results in a transient, rapidly desensitizing GABAergic conductance; whereas, tonic inhibition is mediated by extrasynaptic receptors with a high affinity for GABA and results in a persistent GABAergic conductance. The specific functions of tonic versus phasic GABAergic inhibition in different cell types and the impact on specific neural circuits are only beginning to be unraveled. Here we review the diversity in the magnitude of tonic GABAergic inhibition in various brain regions and cell types, and highlight the impact on neuronal excitability in different neuronal circuits. Further, we discuss the relevance of tonic inhibition in various physiological and pathological contexts as well as the potential of targeting these receptor subtypes for treatment of diseases, such as epilepsy.

## Introduction

Effective communication between neurons requires coordination of synaptic transmission, involving the regulation of neuronal excitability through inhibitory synaptic transmission. In the central nervous system (CNS), this inhibition occurs primarily through GABAergic signaling onto ionotropic GABA_A_ receptors (GABA_A_Rs), which typically provide an inward chloride conductance that hyperpolarizes the cell. The rapid inhibitory actions of GABA are mediated by GABA_A_Rs, which are heteropentamers assembled from 19 identified subunits, but typically consist of two α subunits, two β subunits, and a variable fifth subunit (γ, δ, ε, θ, or π) (for review see Olsen and Sieghart 2008). The functional properties of a receptor are conferred by its specific subunit composition. However, the possible receptor combinations are limited in part due to the differences in the distribution of the individual subunits.

### Regional variations in GABA_A_R subunit expression

Several studies have characterized the patterns of GABA_A_R subunit expression at both the mRNA and protein levels in the rodent brain (Laurie et al., 1992a; Persohn et al., 1992; Wisden et al., 1992; Fritschy and Mohler, 1995; Sperk et al., 1997; Pirker et al., 2000; Schwarzer et al., 2001; Hortnagl et al., 2013), allowing relative comparisons across brain regions for each subunit (for review see Uusi-Oukari and Korpi 2010). From these findings, it can be seen that while some subunits are widely expressed throughout the brain, others have more restricted and distinct distributions. Unless otherwise mentioned, the following distributions of GABA_A_R subunit expression are summarized from those studies in which comparison across brain regions was possible.

The GABA_A_R α1 subunit is expressed at high levels in the olfactory bulb, cortex, thalamus, cerebellum, and hippocampus, and lower levels in the bed nucleus of the stria terminalis (BnST), amygdala, and hypothalamus. The α2 subunit is highly expressed in the cortex, striatum, olfactory bulb, amygdala, hippocampus, and BnST, with lower expression in the hypothalamus, thalamus, and cerebellum. Although the α1 and α2 subunits are both widely expressed and have overlapping patterns, there is an inverse relationship in their densities of expression. The GABA_A_R α3 subunit is primarily expressed in the cortex, olfactory bulb, and thalamus. The α4 subunit is highly expressed in the thalamus, hippocampus, striatum, and cortex. Expression of the α5 subunit is restricted to high expression in the hippocampus and lower expression in the cortex, olfactory bulb, and hypothalamus. Similarly, the expression of the GABA_A_R α6 subunit is the most restricted, with expression almost entirely limited to the cerebellum. The GABA_A_R β 1 subunit is highly expressed in the cortex, hippocampus, and hypothalamus, while it is expressed at lower levels in the thalamus, cerebellum, and amygdala. The expression pattern of the β 2 subunit overlaps with the α1 subunit and is widely expressed in the brain, with the highest levels in the olfactory bulb, cortex, hippocampus, thalamus, striatum, and cerebellum. The β 3 subunit is also highly expressed in the brain, distributed in the cortex, hippocampus, striatum, olfactory bulb, and cerebellum.

The GABA_A_R γ1 subunit has limited expression in the brain, with minimal expression in the hippocampus, BnST, amygdala, and striatum. In contrast, the γ2 subunit is highly expressed in the brain and expression is detected in the olfactory bulb, hippocampus, cortex, hypothalamus, cerebellum, striatum, thalamus, BnST, and amygdala. Expression of the γ3 subunit is also limited, with expression in the cortex and thalamus. Expression of the GABA_A_R δ subunit has been identified in the cortex, hippocampus, thalamus, striatum, and cerebellum. The distributions of ε and θ are overlapping and highly restricted, with expression predominantly in the brainstem and hypothalamus (Moragues et al., 2000, 2002). Expression of the π subunit has not been observed in the brain, but is present in the lung, thymus, and prostate, and is especially abundant in the uterus (Hedblom and Kirkness, 1997). Finally, the ρ 1, ρ 2, and ρ 3 subunits are expressed in the cerebellum and retina, while ρ 3 is also expressed in the hippocampus (Boue-Grabot et al., 1998; Martinez-Delgado et al., 2010). These ρ subunits can assemble into heteromeric or homomeric receptors with distinct kinetics and pharmacological properties from typical GABA_A_Rs (for review see Martinez-Delgado et al. 2010).

### Diversity of GABA_A_R subunit configurations

Although there are 19 subunits, the heterogeneous distribution of GABA_A_R subunits limits the possible configurations. In addition, there is selective partnership of GABA_A_R subunits, in which certain subunits preferentially assemble together. For example, the α1 subunit appears to partner with the β 2/3 and γ2 subunits (Benke et al., 1991), whereas the δ subunit preferentially partners with α4/6 and β 2/3 subunits (Tretter et al., 2001; Korpi et al., 2002). The combination of the regional distribution and selective partnership of subunits results in different configurations of GABA_A_Rs localized in different brain regions (for review see Olsen and Sieghart 2008). For example, GABA_A_Rs composed of α6β 2/3δ are expressed exclusively in the cerebellum (Thompson et al., 1992; Nusser et al., 1999), while the α4β 2/3δ combination is expressed in the dentate gyrus of the hippocampus (Peng et al., 2002). The unique patterns of expression and rules of partnership of the different GABA_A_R subunits in the brain suggest diversity in GABAergic inhibition in distinct brain regions and cell types.

GABA_A_Rs with unique subunit assemblies not only have differences in regional distribution, but the subunit composition also dictates the subcellular localization of these receptors. GABA_A_Rs containing the α1 and γ2 subunits are localized synaptically, whereas α4, α5, α6, and δ subunits are predominantly localized perisynaptically or extrasynaptically (Farrant and Nusser, 2005). Interestingly, the GABA_A_R α2 subunit is localized to the axon initial segment (AIS) (Nusser et al., 1996). However, this distribution may vary between cell types. For example, GABA_A_R δ subunit immunostaining has been demonstrated in the perikarya of interneurons (Pirker et al., 2000; Wei et al., 2003), while in principal neurons, such as dentate gyrus granule cells, the distribution of this subunit is restricted to the dendrites. The subcellular distribution of the specific subtypes of GABA_A_Rs provides information regarding the function of these receptors. For example, receptors located synaptically mediate the phasic form of GABAergic inhibition; whereas, those located extrasynaptically mediate the tonic form of GABAergic inhibition (for review see Farrant and Nusser 2005), Brickley and Mody (2012)]. Phasic inhibition is mediated by synaptically-clustered receptors responding to the release of GABA across the synaptic cleft. Activation of these low-affinity receptors results in a transient, rapidly-desensitizing postsynaptic response. In contrast, tonic inhibition is mediated by extrasynaptic receptors with a high affinity for GABA (Stell and Mody, 2002). These receptors provide a persistent conductance that is revealed by the application of a high concentration of GABA_A_R antagonists. These unique properties of synaptic and extrasynaptic GABA_A_Rs are conferred by their specific subunit composition, which dictates the GABA binding affinity, kinetics, and subcellular localization (Farrant and Nusser, 2005; Brickley and Mody, 2012). This diversity of receptor configurations results in variations in GABAergic conductances across numerous brain regions and cell types.

### GABA_A_R ligands and subunit composition

Besides influencing how GABA_A_Rs respond to its neurotransmitter, subunit composition also determines how the receptors can be modulated by other ligands. For example, receptors that contain the γ2 subunit exhibit sensitivity to benzodiazepines (Pritchett et al., 1989; Gunther et al., 1995), which bind at the interface of the γ2 subunit and an α1, α2, α3, or α5 subunit. The distinct actions of benzodiazepines are mediated by the specific α subunit expressed in different regions of the brain (Rudolph et al., 1999). The specific subunit combinations that mediate the effects of various anesthetics are also being investigated (Belelli et al., 2009; Brickley and Mody, 2012; Houston et al., 2012). In addition, glycine and taurine have been shown to influence tonic inhibition, but the effects of these ligands are beyond the scope of this review.

Neurosteroids comprise an important class of GABA_A_R ligands. These neuroactive metabolites, whether synthesized in the brain or derived from peripheral steroids, can act as positive or negative allosteric modulators of GABA_A_R function in a number of physiological and pathological contexts (for review see MacKenzie and Maguire 2013). Slight changes in neurosteroid levels can greatly impact the GABAergic regulation of neuronal excitability. The δ subunit has been shown to confer enhanced sensitivity to modulation by neurosteroids (Wohlfarth et al., 2002) despite the fact that the neurosteroid binding site has been identified on the α/β interface (Hosie et al., 2006, 2009). However, other GABA_A_R combinations are also sensitive to neurosteroid modulation at higher concentrations (Stell et al., 2003; Belelli et al., 2009). As such, neurosteroids have the potential to exert different effects across multiple brain regions.

Beyond subunit composition and the presence of other ligands, the actions of GABA_A_Rs depend on ion gradients, transporter function, as well as other factors that affect the local environment. Thus, the nature of GABAergic transmission can be highly variable. The impact of these factors on GABAergic inhibition in specific brain regions and cell types can be difficult to assess, mainly due to technical limitations, including the use of standard intracellular solutions which disrupt native ionic concentration gradients and the use of the acute slice preparation in which endogenous ligands may be washed out. However, we are still able to compare the magnitude of GABAergic inhibition in different brain regions and cell types when taking into consideration the experimental methods. In this review, we summarize the differences in the magnitude of tonic GABAergic inhibition across various brain regions and cell types, and compare its impact on neuronal excitability. We discuss the role of tonic inhibition in both physiological and pathological conditions, and explore the therapeutic potential of targeting specific receptor subtypes for treatment of diseases such as epilepsy.

## Tonic inhibition varies by brain region

### Hippocampus

The trisynaptic circuit of the hippocampus includes the perforant path input from the entorhinal cortex to granule cells of the dentate gyrus, which send mossy fibers to pyramidal neurons in the CA3 subfield, which send Schaffer collateral projections to pyramidal neurons in CA1, which then send projections back to the entorhinal cortex. These recurrent connections create the potential for seizure generation. Thus, GABAergic inhibition is paramount in controlling neuronal excitability in the hippocampus. Tonic GABAergic inhibition plays a critical role in regulating the excitability of the hippocampus. This section will review the role of tonic GABAergic inhibition in the different hippocampal subregions.

#### Dentate gyrus

The dentate gyrus has been termed the “dentate gate” due to its role in regulating the excitability of the hippocampal network (Coulter and Carlson, 2007). Tonic GABAergic inhibition has been proposed to maintain the integrity of the dentate gate (Coulter and Carlson, 2007). Dentate gyrus granule cells (DGGCs) exhibit low firing rates and their resting membrane potential is hyperpolarized compared to other principal neurons (Scharfman and Schwartzkroin, 1989; Jung and McNaughton, 1993). Early patch-clamp studies in the hippocampus showed the existence of a background GABAergic conductance that was not dependent upon action potentials (Otis et al., 1991). Later, it was demonstrated that this tonic GABAergic current in DGGCs is primarily mediated by δ subunit-containing receptors (Stell et al., 2003). Although the tonic GABAergic inhibition in DGGCs is largely mediated by δ subunit-containing GABA_A_Rs, a role for the α5 subunit also been demonstrated (Glykys et al., 2008; Herd et al., 2008). Immunohistochemical and electron microscopy studies have determined that δ subunit-containing receptors are localized to perisynaptic zones on the dendrites of DGGCs in the molecular layer, suggesting activation by spillover of synaptic GABA (Wei et al., 2003). Consistent with this hypothesis, the magnitude of tonic GABAergic inhibition is increased in the presence of the GABA uptake blockers (Table 1) (compare Nusser and Mody, 2002; Stell and Mody, 2002; Stell et al., 2003; Pandit et al., 2013 with Herd et al., 2008; Zhan and Nadler, 2009; Gupta et al., 2012). Studies have determined that in the hippocampus, the ambient GABA acting on extrasynaptic GABA_A_Rs comes from synaptic release (Glykys and Mody, 2007). However, GABA has also been shown to be released from glial cells (Kozlov et al., 2006; Lee et al., 2010b) and dendrites (Zilberter et al., 1999), and in both synaptic and non-synaptic fashions (Olah et al., 2009). Interestingly, distinct GABA uptake mechanisms may regulate the ambient GABA levels arising from these different sources (Song et al., 2013). Insight into the mechanisms controlling GABA release and uptake may have a significant impact on our understanding of the activity of neural circuits.

**Table 1:** **Levels of tonic inhibition vary across brain region and cell type in the CNS**.

**Region and cell type^a^**	**Tonic conductance^b^**	**Experimental procedures^c^ (concentration in μM)**	**GABA_A_R subunits**	**References**
**HIPPOCAMPUS**				
Dentate gyrus				
*Granule cells*	12.8 ± 3.0 pA	BIC (100–150) or GBZ (100–150)	α4	Nusser and Mody, 2002
	47.6 ± 10.1 pA	NO-711 (2.5) + BIC (100–150) or GBZ (100–150)		
		Rat (Wistar), >3 month; high Cl^−^ internal; 34–36°C; KYN (3000–5000)		
	7.4 ± 1.1 pA	NO-711 (10) + GBZ (100–200); 13–29 day male	δ	Stell and Mody, 2002; Stell et al., 2003
	43.8 ± 10.8 pS/pF	NO-711 (10) + GBZ (100–200); 30–181 day male; –60 mV		
		Mouse (C57Bl/6); high Cl^−^ internal; 33–35°C; KYN (3000–5000)		
	172.4 ± 51.4 pS/pF	GABA (5) + GBZ (>100); male	δ	Maguire et al., 2005
	29.8 ± 5.5 pS/pF	GABA (5) + GBZ (>100); 10–14 week female, estrus		
	57.6 ± 10.2 pS/pF	GABA (5) + GBZ (>100); 10–14 week female, diestrus		
		Mouse (C57Bl/6); −60 mV; high Cl^−^ internal; KYN (3000)		
	72 ± 19 pA n.s.	BIC (100)		Mtchedlishvili and Kapur, 2006
		GBZ (100)		
		Rat (Sprague–Dawley), adult male; −65 mV; high Cl^−^ internal; 20–22°C; AP5 (50), CNQX (20)		
	29.5 ± 5.4 pA	GABA (5) + GBZ (>100)	δ, γ	Zhang et al., 2007
		Mouse (C57Bl/6), 30–60 day male; −70 mV; high Cl^−^ internal; 35°C; KYN (3000–5000)		
	22.0 ± 5.33 pA	GABA (5) + BIC	δ, α5	Glykys et al., 2008
		Mouse (C57Bl/6), >35d male; −70 mV; high Cl^−^ internal; 32–34°C; KYN (3000)		
	16.6 ± 2.7 pA	BIC (30)	α4β2δ, α5β1/3γ2	Herd et al., 2008
		Mouse (mixed), 20–26 day; −60 mV; high Cl^−^ internal; 35°C; KYN (2000), TTX (0.5)		
	1.4 ± 0.3 pA	GBZ (100); rat (Sprague–Dawley), male		Zhan and Nadler, 2009
	0.5 ± 0.2 pA	GBZ (100); mouse (C57Bl/6), >18 week male		
		−70 mV; high Cl^−^ internal; 34–35°C; AP5 (50), NBQX (10)		
	8.4 ± 1.1 pA	BIC (100)	α4βδ	Gupta et al., 2012
	0.15 ± 0.03 pA/pF	Rat (Wistar), 4–5 week male; –70 mV; high Cl^−^ internal; 33°C; KYN (3000)		
	11.9 ± 1.5 pA	PTX (100)		Wlodarczyk et al., 2013
	10.6 ± 3.6 pA	PTZ (1500)		
	5.9 ± 0.6 pA	BIC (10)		
	−6.7 ± 1.6 pA	GBZ (125)		
		Rat (Sprague–Dawley), 3–4 week; –70 mV; high Cl^−^ internal; 35°C; AP5 (50), NBQX (20), MCPG (250), CGP55845 (1)		
	3.36 ± 0.43 pA	BIC (20); Wistar	δ	Pandit et al., 2013
	1.74 ± 0.35 pA	BIC (20); Noda epileptic rat		
	17.32 ± 3.56 pA	GABA (3) + BIC (20); Wistar		
	8.86 ± 1.55 pA	GABA (3) + BIC (20); Noda epileptic rat		
	28.28 ± 3.53 pA	NO-711 (5) + BIC (20); Wistar		
	14.56 ± 3.41 pA	NO-711 (5) + BIC (20); Noda epileptic rat		
		Rat, 8 week male; –70 mV; high Cl^−^ internal; 32°C; AP5 (100), CNQX (10)		
	5.5 ± 1.2 pA	GBZ (>200); control	δ	Lee and Maguire, 2013
	5.6 ± 0.7 pA	GBZ (>200); *Gabrd/Gad*		
		Mouse, 8–12 week; −70 mV; high Cl^−^ internal; 33°C; KYN (3000)		
*Semilunar granule cells*	16.7 ± 1.7 pA	BIC (100); –70 mV	δ	Gupta et al., 2012
	0.18 ± 0.04 pA/pF	BIC (100); 0 mV		
	22.5 ± 6.7 pA	Rat (Wistar); high Cl^−^ internal; 33°C; KYN (3000)		
*ML interneurons*	9.81 ± 1.51 pA	GABA (5) + BIC (100-200)	α1βδ	Glykys et al., 2007
		Mouse (C57/Bl6), 1–4 month male; –70 mV; high Cl^−^ internal; 32–34°C; KYN (3000)		
	12.2 ± 1.68 pA	GABA (5) + BIC	δ, α5	Glykys et al., 2008
		Mouse (C57Bl/6), >35d male; –70 mV; high Cl^−^ internal; 32–34°C; KYN (3000)		
	13.3 ± 1.9 pA	GBZ (>200); control	δ	Lee and Maguire, 2013
	3.5 ± 1.3 pA	GBZ (>200); *Gabrd/Gad*		
		Mouse, 8–12 week; −70 mV; high Cl^−^ internal; 33°C; KYN (3000)		
Hilus				
*FS basket cells*	5.7 ± 0.9 pA	GBZ (10)	δ	Yu et al., 2013
		Rat (Wistar), 4–5 week male; –70 mV; high Cl^−^ internal; 33°C; KYN (3000)		
*Non-FS interneurons*	0.5 ± 0.9 pA, n.s.	GBZ (10)		Yu et al., 2013
		Rat (Wistar), 4–5 week male; –70 mV; high Cl^−^ internal; 33°C; KYN (3000)		
CA3				
*Pyramidal cells*	30.1 ± 5.28 pA	GABA (5) + BIC	δ, α5	Glykys et al., 2008
		Mouse (C57Bl/6), >35 day male; –70 mV; high Cl^−^ internal; 32–34°C; KYN (3000)		
*Interneurons*	18.8 ± 4.2 pA	GBZ (20)	δ	Mann and Mody, 2010
		Mouse (C57Bl/6), 4–12 week male; –70 mV; high Cl^−^ internal; 32–34°C; KYN (3000)		
CA1				
*Pyramidal cells*	35.1 ± 9.9 pA	BIC (10)		Bai et al., 2001
	3.5 ± 1.7 pA, n.s.	GBZ (20)		
		Rat (Wistar), 2–3 week; high Cl^−^ internal; −60 mV; 31°C; AP5 (40), CNQX (10), TTX (0.5)		
	−0.9 ± 1.5 pA, n.s.	PTX (100)		Semyanov et al., 2003
	−0.2 ± 3.0 pA, n.s.	BIC (10)		
	−3.6 ± 3.7 pA, n.s.	GBZ (0.5)		
		Guinea pig, 3–4 week; –60 mV; high Cl^−^ internal; 23–25°C; KYN (3000), NBQX (50), AP5 (50), MSOP (100), CGP52432 (5)		
	34.5 ± 9.6 pA	BIC (100)	α5	Caraiscos et al., 2004
		Mouse (mixed), 18–23 day male; –60 mV; high Cl^−^ internal; 35°C; KYN (2000)		
	17.1 ± 5.5 pA	PTX (100)	δ	Scimemi et al., 2005
		Rat (Sprague–Dawley), 8–10 week male; –60 mV; high Cl^−^ internal; 23–25°C; NBQX (25), AP5 (50), CGP52432 (5)		
	38.9 ± 5.10 pA	GABA (5) + BIC	α5, δ	Glykys et al., 2008
		Mouse (C57Bl/6), >35 day male; –70 mV; high Cl^−^ internal; 32–34°C; KYN (3000)		
	8.7 ± 2.4 pA	GBZ (>200); control	δ	Lee and Maguire, 2013
	26.5 ± 6.7 pA	GBZ (>200); *Gabrd/Gad*		
		Mouse, 8–12 week; –70 mV; high Cl^−^ internal; 33°C; KYN (3000)		
*Str. radiatum interneurons*	16 ± 4 pA	PTX (100)	γ	Semyanov et al., 2003
	21 ± 10 pA	BIC (10)		
	−5.6 ± 5.7 pA, n.s.	GBZ (0.5)		
		Guinea pig, 3–4 week; −60 mV; high Cl^−^ internal; 23–25°C; KYN (3000), NBQX (50), AP5 (50), MSOP (100), CGP52432 (5)		
	0.62 ± 0.16 nS	PTX (100)		Song et al., 2011
		Mouse (C57Bl/6), 4–6 week male; high Cl^−^ internal; 34°C; NBQX (25), AP5 (50), CGP52432 (5)		
	23.2 ± 6.1 pA	GBZ (>200); control	δ	Lee and Maguire, 2013
	3.7 ± 1.2 pA	GBZ (>200); *Gabrd/Gad*		
		Mouse, 8–12 week; –70 mV; high Cl^−^ internal; 33°C; KYN (3000)		
*Str. oriens interneurons*	30 ± 5 pA	PTX (100)	γ	Semyanov et al., 2003
		Guinea pig, 3–4 week; –60 mV; high Cl^−^ internal; 23–25°C; KYN (3000), NBQX (50), AP5 (50), MSOP (100), CGP52432 (5)		
Subiculum				
*Pyramidal neurons*	5.32 ± 0.88 pA/pF	PTX (100)	α5, δ	Curia et al., 2009
		Mouse (C57Bl/6), 4–24 week male; –70 mV; high Cl^−^ internal; room temperature; CPP (10), CNQX (10), KYN (2000), CGP55845 (4)		
*Intrinsically bursting cells*	1.75 ± 0.27 pA/pF	PTX (100)		Panuccio et al., 2012
		Rat (Sprague–Dawley), 2–3 month male; –70 mV; high Cl^−^ internal; 21°C; CNQX (10), CPP (10), CGP55845 (4)		
*Regular spiking cells*	0.58 ± 0.16 pA/pF	PTX (100)		Panuccio et al., 2012
		Rat (Sprague–Dawley), 2–3 month male; –70 mV; high Cl^−^ internal; 21°C; CNQX (10), CPP (10), CGP55845 (4)		
*Interneurons*	0.6 ± 0.12 pA/pF	PTX (100)		Panuccio et al., 2012
		Rat (Sprague–Dawley), 2–3 month male; –70 mV; high Cl^−^ internal; 21°C; CNQX (10), CPP (10), CGP55845 (4)		
**CEREBELLUM**				
*Granule cells*	100 ± 16 pS	BIC (10)		Brickley et al., 1996
		Rat (Sprague–Dawley), 14 day; –70 mV; high Cl^−^ internal; 22–25°C; CNQX (5), AP5 (10), strychnine (0.3)		
	0.01 ± 0.3 pA/pF	BIC (10); 7–8 day male		Wall and Usowicz, 1997
	3.5 ± 0.6 pA/pF	BIC (10); 10–14 day male		
	3.4 ± 0.5 pA/pF	BIC (10); 20–25 day male		
	8.7 ± 1.3 pA/pF	BIC (10); 40–49 day male		
		Rat (Wistar); –70 mV; high Cl^−^ internal; 22–24°C		
	~20 pS/pF	GBZ (10); 7 day male	α6	Brickley et al., 2001
	~60 pS/pF	GBZ (10); 14 day male		
	~130 pS/pF	GBZ (10); 35 day male		
		Mouse (mixed); –70 mV; high Cl^−^ internal; 23–25°C		
	49.7 ± 7.5 pS/pF	NO-711 (10) + GBZ (100–200)	δ	Stell et al., 2003
		Mouse (C57Bl/6), 30–181 day male; –70 mV; high Cl^−^ internal; 20–22°C; KYN (3000–5000)		
	126 ± 24 pS/pF	GBZ (40)		Houston et al., 2012
		Mouse (C57Bl/6), 4–6 week male; –60 mV; high Cl^−^ internal; room temperature; CNQX (5)		
*Purkinje cells*	n.s.	BIC (10)		Wall and Usowicz, 1997
		Rat (Wistar), adult male; –70 mV; high Cl^−^ internal; 22–24°C		
	n.s.	BIC		Harvey et al., 2006
	n.s.	TPMPA		
	48 ± 10 pA	NO-711 (40) + SNAP-5114 (50) + BIC (10) Mouse (TO), 3–5 week male; –70 mV; high Cl^−^ internal; 20–24°C; CGP55845		
**CORTEX**				
Layer 1				
*Interneurons*	~50 pA	BIC (20)		Keros and Hablitz, 2005
		Rat (Sprague–Dawley), 17–22 day male; –65 mV; high Cl^−^ internal; 32–35°C; CNQX (10), AP5 (20), SCH50911 (10)		
Layer 2/3				
*Pyramidal cells*				
(frontoparietal cortex)	1.2 ± 0.9 pA	GBZ (>100)	α4βδ	Drasbek and Jensen, 2006; Drasbek et al., 2007
	26.6 ± 3.6 pA	GABA (0.8) + NO-711 (10) + GBZ (>100)		
	43.2 ± 4.7 pA	THIP (1) + GBZ (>100)		
		Mouse (C57Bl/6), 13–19 day male; –70 mV; high Cl^−^ internal; 33–34°C; KYN (3000)		
(frontoparietal cortex)	66.2 ± 19.0 pA	THIP (1) + GBZ (>100)		Vardya et al., 2008
	26.5 ± 3.5 pA	GABA (0.8) + NO-711 (10) + PTX (100)		
		Mouse (SST-GFP), 14–24 day male; –70 mV; high Cl^−^ internal; 33°C		
(somatosensory cortex)	2.2 ± 0.8 pA	GBZ (0.5)		Bragina et al., 2008
	4.3 ± 1.1 pA	PTX (100)		
		Mouse (C57Bl/6), 2 month; –70 mV; high Cl^−^ internal; 33–34°C; DNQX (20), CPG55845 (1)		
(motor cortex)	8.05 ± 0.80 pA/pF	GBZ (>100); +10 mV	α5, δ	Clarkson et al., 2010
	3.66 ± 1.21 pA/pF	GBZ (>100); –70 mV		
		Mouse (C57Bl/6), 2–4 month male; high Cl^−^ internal; 32–34°C; KYN (3000)		
(visual cortex)	2.4 ± 1.2 pA, n.s.	BIC (10); 3 week		Jang et al., 2010
	12.4 ± 1.2 pA	BIC (10); 5 week		
	10.1 ± 1.3 pA	BIC (10); 8 week		
		Rat (Sprague–Dawley); –75 mV; high Cl^−^ internal; 32–33°C; DNQX (20), AP5 (50), CGP52432 (1)		
*SST + interneurons* (frontoparietal cortex)	7.8 ± 0.8 pA	THIP (1) + GBZ (>100)		Vardya et al., 2008
	5.6 ± 2.3 pA	GABA (0.8) + NO-711 (10) + PTX (100)		
		Mouse (SST-GFP), 14–24 day male; –70 mV; high Cl^−^ internal; 33°C		
*Neurogliaform cells* (somatosensory cortex)	8 ± 3 pA	GABA (5) + GBZ (10)	δ	Olah et al., 2009
		Rat (Wistar), 22–35 day; –51 mV; low Cl^−^ internal; 35°C; CGP35348 (40)		
Layer 4 (barrel cortex)				
*Excitatory RS neurons*	0.20 ± 0.02 pA/pF	PTX (100)	δ	Urban-Ciecko et al., 2010
		Mouse (Swiss), 5–7 week female; –75 mV; high Cl^−^ internal; 32°C		
*RS/LTS interneurons*	0.71 ± 0.09 pA/pF	PTX (100)	δ	Urban-Ciecko et al., 2010
		Mouse (Swiss), 5–7 week female; –75 mV; high Cl^−^ internal; 32°C		
*FS interneurons*	1.3 ± 0.13 pA/pF	PTX (100)	δ	Urban-Ciecko et al., 2010
		Mouse (Swiss), 5–7 week female; –75 mV; high Cl^−^ internal; 32°C		
Layer 5				
*Pyramidal cells*				
(frontoparietal cortex)	30.1 ± 3.5 pA	THIP (1) + GBZ (>100)	δ	Drasbek and Jensen, 2006
		Mouse (C57Bl/6), 13–19 day male; –70 mV; high Cl^−^ internal; 33–34°C; KYN (3000)		
(frontal cortex)	24.4 ± 6.5 pA	PTX (50); WT		Nishikawa et al., 2011
	13.3 ± 5.5 pA	PTX (50); GAD65^−/−^		
		Mouse, 12–16 week male; 0 mV; low Cl^−^ internal; 22–24°C		
**AMYGDALA**				
BLA				
*Pyramidal cells*	18.6 ± 2.9 pA	BIC (10)		Wu et al., 2007
		Mouse (C57Bl/6), 6–10 week male; –70 mV; low Cl^−^ internal; room temperature		
	20.0 ± 5.2 pA	GBZ (100)	δ	Olmos-Serrano et al., 2010
		Mouse (FVB), 20–30 day male; –60 mV; high Cl^−^ internal; 21–23°C; DNQX (20), AP5 (50), baclofen (1)		
	0.215 ± 0.024 pA/pF	BIC (25)	α3βγ2	Marowsky et al., 2012
		Mouse (C57Bl/6), 21–49 day male; –70 mV; high Cl^−^ internal; 32–34°C; KYN (2500), CGP54626 (0.5)		
LA				
*Pyramidal cells*	0.105 ± 0.027 pA/pF	BIC (25)		Marowsky et al., 2012
	(70%)	Mouse (C57Bl/6), 21–49 day male; –70 mV; high Cl^−^ internal; 32–34°C; KYN (2500), CGP54626 (0.5)		
Interneurons				
*LA/BLA interneurons*	0.143 ± 0.014 pA/pF	BIC (25)		Marowsky et al., 2012
		Mouse (GAD67-GFP), 21–49 day male; –70 mV; high Cl^−^ internal; 32–34°C; KYN (2500), CGP54626 (0.5)		
*Paracapsular cells*	0.160 ± 0.02 pA/pF	BIC (25)		Marowsky et al., 2012
		Mouse (GAD67-GFP), 21–49 day male; –70 mV; high Cl^−^ internal; 32–34°C; KYN (2500), CGP54626 (0.5)		
CeA				
*CRF1*+ *cells*	19.7 ± 2.4 pA	GBZ (100)	α1	Herman et al., 2013
	19.0 ± 3.1 pA	PTX (100)		
		Mouse (CRF1-GFP), 2–6 month male; –60 mV; high Cl^−^ internal; room temperature; DNQX (20), AP5 (50), CGP55845 (1)		
*CRF1*− *cells*	−1.8 ± 2.7 pA, n.s.	GBZ (100)	δ	Herman et al., 2013
		Mouse (CRF1-GFP), 2–6 month male; –60 mV; high Cl^−^ internal; room temperature; DNQX (20), AP5 (50), CGP55845 (1)		
**STRIATUM**				
*D1*+ *cells*				
*Juvenile*	1.7 ± 0.8 pA	BIC (25); 22–24°C	α5β3γ2	Ade et al., 2008; Janssen et al., 2009
	3.0 ± 1.7 pA	GBZ (10); 22–24°C		
	1.6 ± 1.3 pA	BIC (25) or GBZ (10); 31°C		
		Mouse (D2-EGFP), 15–25 day; –70 mV; high Cl^−^ internal		
*Adult*	36.5 ± 8.9 pA	GABA (5) + BIC (100)	δ	Santhakumar et al., 2010
		Mouse (D2-EGFP), >30 day male; –70 mV; high Cl^−^ internal; 34°C; AP5 (10), DNQX (25)		
*D2*+ *cells*				
*Juvenile*	18.9 ± 0.3 pA	BIC (25); 22–24°C	α5β3γ2	Ade et al., 2008; Janssen et al., 2009
	16.0 ± 1.2 pA	GBZ (10); 22–24°C		
	30.4 ± 5.5 pA	BIC (25) or GBZ (10); 31°C		
		Mouse (D2-EGFP), 15–25 day; –70 mV; high Cl^−^ internal		
*Adult*	6.2 ± 2.0 pA	GABA (5) + BIC (100)	δ	Santhakumar et al., 2010
		Mouse (D2-EGFP), >30 day male; –70 mV; high Cl^−^ internal; 34°C; AP5 (10), DNQX (25)		
**THALAMUS**				
VB				
*TC neurons*	16.5 ± 2.0 pA	BIC (20)	α4β2δ	Jia et al., 2005
		Mouse (C57Bl/6), 12–20 day; –65 mV; high Cl^−^ internal; 20–22°C; KYN (3000–5000)		
	17.6 ± 13.4 pA (62.5%)	GBZ (50)		Cope et al., 2005
		Rat (Wistar), 14–21day; –70 mV; high Cl^−^ internal; 32°C; KYN (3000)		
RTN	n.s.	BIC (20)		Jia et al., 2005
		Mouse (C57Bl/6), 12–20 day; –65 mV; high Cl^−^ internal; 20–22°C; KYN (3000–5000)		
dLGN				
*TC neurons*	16.4 ± 13.7 pA (75%)	GBZ (50)	δ	Cope et al., 2005
		Rat (Wistar), 14–21 day; –70 mV; high Cl^−^ internal; 32°C; KYN (3000)		
	54.2 ± 13.9 pS/pF	GBZ (20)	δ	Bright et al., 2007
		Mouse (C57Bl/6), >1 month; –60 mV; high Cl^−^ internal; 35–38°C; KYN (500)		
*Interneurons*	6.7 ± 4.5 pS/pF, n.s.	GBZ (20)		Bright et al., 2007
		Mouse (C57Bl/6), >1 month; –60 mV; high Cl^−^ internal; 35–38°C; KYN (500)		
vLGN				
*TC neurons*	6.6 ± 4.1 pS/pF, n.s.	GBZ (20)		Bright et al., 2007
		Mouse (C57Bl/6), >1 month; –60 mV; high Cl^−^ internal; 35–38°C; KYN (500)		
*Interneurons*	2.5 ± 0.9 pS/pF, n.s.	GBZ (20)		Bright et al., 2007
		Mouse (C57Bl/6), >1 month; –60 mV; high Cl^−^ internal; 35–38°C; KYN (500)		
dMGB	~22 pA	GBZ (50); 3–8 month	α4δ	Richardson et al., 2013
	~9 pA	GBZ (50); 28–32 month		
		Rat (FBN), male; –60 mV; high Cl^−^ internal; 22°C; DNQX (10), AP5 (50)		
vMGB	~25 pA	GBZ (50); 3–8 month	α4δ	Richardson et al., 2013
	~6 pA	GBZ (50); 28–32 month		
		Rat (FBN), male; –60 mV; high Cl^−^ internal; 22°C; DNQX (10), AP5 (50)		
**HYPOTHALAMUS**				
SON				
*Oxytocin or vasopressin cells*	20.20 ± 2.54 pA	BIC (20) or PTX (100–300); 22–24°C	α5βγ2	Park et al., 2006; Jo et al., 2011
	19.8 ± 2.7 pA	BIC (20) or PTX (100–300); 35°C		
	n.s.	GBZ (1–300); 22–24°C		
		Rat (Wistar, Sprague–Dawley), >5 week male; –70 mV; high Cl^−^ internal; AP5 (100), CNQX (10)		
PVN				
*RVLM-projecting neurons*	9.79 ± 1.28 pA	BIC (20); room temperature	δ	Park et al., 2007, 2009
	11.38 ± 0.57 pA	BIC (20); 35°C		
	n.s.	GBZ (0.1–100)		
		Rat (Wistar), male; –70 mV; high Cl^−^ internal		
*CRH neurons*	11.2 ± 2.5 pA	GBZ (>200)	δ	Sarkar et al., 2011
	20.9 ± 2.5 pA	THDOC (0.01) + GBZ (>200)		
		Mouse (C57Bl/6, CRH-GFP), 3 month male; –70 mV; high Cl^−^ internal; 33°C; KYN (3000)		
	13.8 ± 3.4 pA	GBZ (>200); control	δ	Lee et al., 2013
	0.7 ± 0.2 pA	GBZ (>200); *Gabrd/Crh*		
		Mouse, 8–12 week male; –70 mV; high Cl^−^ internal; 33°C; KYN (3000)		
Preoptic area	7.7 ± 4.3 pA	L-655, 708 (50); male	α5	Penatti et al., 2009a,b
	10.4 ± 2.0 pA (73%)	L-655, 708 (50); female		
		Mouse (C57Bl/6), 13 week; –70 mV; high Cl^−^ internal; 20–22°C; KYN (2000)		
*GnRH neurons*	3.83 ± 0.97 pA	PTX (100); male		Penatti et al., 2010, 2011
	~3 pA (50%)	PTX (100); female		
		Mouse (GnRH-GFP), 8 week; –70 mV; high Cl^−^ internal; 20–22°C; KYN (2000)		
	17.0 ± 3.2 pA (56%)	PTX (300)	δ	Bhattarai et al., 2011
	13.1 ± 3.1 pA (44%)	BIC (20)		
		Mouse (GnRH-GFP), 6–76 day; –60 mV; high Cl^−^ internal; room temperature; KYN (2000)		
TMN				
*Histaminergic neurons*	n.s.	BIC (20)		Zecharia et al., 2009
		GBZ (20)		
		Mouse, 8–12 week male; –60 mV; high Cl^−^ internal; 21–22°C; KYN (1000), strychnine (1000)		
Perifornical area				
*Orexinergic neurons*	n.s.	BIC (20)		Zecharia et al., 2009
		GBZ (20)		
		Mouse, 8–12 week male; –60 mV; high Cl^−^ internal; 21–22°C; KYN (1000), strychnine (1000)		
**MEDULLA**				
*Hypoglossal motoneurons*	9.2 ± 4.7 pA, n.s.	GBZ (20)	δ	Numata et al., 2012
	104.3 ± 20.9 pA	GABA (5) + SKF89976A (30) + SNAP-5114 (50) + BIC (20)		
		Mouse, 3–15 day; –70 mV; high Cl^−^ internal; 30°C; DNQX (10), AP5 (50), strychnine (1), CGP55845 (1), TTX (1)		
	20.9 ± 2.3 pA	BIC (100); –70 mV, high Cl^−^ internal	αβδ, αβε	Chesnoy-Marchais, 2013
	26.0 ± 12.7 pA	BIC (100); –10 mV, low Cl^−^ internal		
	26.1 ± 7.0 pA	PTX (100); –70 mV, high Cl^−^ internal		
		Rat (Sprague–Dawley), 11–15 day; 23–26°C; KYN (1000), strychnine (1)		
**PONS**				
Locus coeruleus				
*Noradrenergic neurons*	n.s.	BIC (20)		Zecharia et al., 2009
		GBZ (20)		
		Mouse, 8–12 week male; –60 mV; high Cl^−^ internal; 21–22°C; KYN (1000), strychnine (1000)		
**SPINAL CORD**				
Dorsal horn				
*Substantia gelatinosa neurons* (lamina II)	650 ± 90 pA/nF (67.1%)	BIC (100)	α5βγ2, α4βδ, αβε	Takahashi et al., 2006
		Mouse (ddY), 3–9 week male; 0 mV; low Cl^−^ internal; 36°C; strychnine (2), TTX (0.5)		
	8.4 ± 0.7 pA	BIC (20)		Ataka and Gu, 2006
		Mouse, 6–9 week; 0 mV; low Cl^−^ internal; 22°C		
	n.s.	BIC (30)		Mitchell et al., 2007
		Rat (Sprague–Dawley), 15–21 day; –60 mV; high Cl^−^ internal; 35°C; KYN (2000), TTX (0.5)		
	1.5 ± 1.3 pA, n.s.	GBZ (1)		Maeda et al., 2010
	8.0 ± 1.1 pA (79%)	BIC (20)		
	9.5 ± 3.2 pA (75%)	GBZ (1) + BIC (20)		
	65.92 ± 21.6 pA	GABA (1000) + BIC (20)		
		Rat (Sprague–Dawley), 6–8 week male; 0 mV; low Cl^−^ internal; 36°C; strychnine (2)		
	0.60 ± 0.16 pA/pF (80%)	GABA (5) + BIC (20)	δ	Bonin et al., 2011
		Mouse, 3–4 month male; 0 mV; high Cl^−^ internal; 36°C; CNQX (10), TTX (0.3)		
*GABA-dominant interneurons* (lamina I/IIo)	~3 pA	BIC (10); 16–18 day		Takazawa and MacDermott, 2010
	~6 pA	BIC (10); 29–32 day		
		Mouse (GAD67-EGFP); 0 mV; low Cl^−^ internal; 32°C		
*Glycine-dominant interneurons* (lamina II/III border)	~6 pA	BIC (10); 16–18 day		Takazawa and MacDermott, 2010
	~1 pA	BIC (10); 29–32 day		
		Mouse (GAD67-EGFP); 0 mV; low Cl^−^ internal; 32°C		
Lateral horn (Intermediolateral cell column)				
*Sympathetic preganglionic neurons*	n.s.	BIC (2–100); resting potential, low Cl^−^ internal	α5βγ2	Wang et al., 2008
	2.4 ± 0.4 mV	BIC (100); resting potential, high Cl^−^ internal		
	4.4 ± 0.5 mV	BIC (100); 0 mV, low Cl^−^ internal		
	4.0 ± 1.4 mV	PTX (100); 0 mV, low Cl^−^ internal		
	n.s.	GBZ (0.025–25); resting potential or 0 mV, low Cl^−^ internal		
		Rat, 10–15 day; room temperature; KYN (2000)		
*Interneurons*	n.s.	BIC (100); 0 mV, low Cl^−^ internal		Wang et al., 2008
		Rat, 10–15 day; room temperature; KYN (2000)		
Ventral horn	9.6 ± 2.8 pA	BIC (25)		Chub and O'Donovan, 2001
		Chick (White Leghorn), E10–11; –70 mV; high Cl^−^ internal; 28°C; TTX (1), AP5 (50), CNQX (10)		
**OLFACTORY BULB**				
*Granule cells*	24.3 ± 10.3 pA	GBZ (500)	α4βδ	Labarrera et al., 2013
		Mouse (C57Bl/6), 25–30 day female; +20 mV; low Cl^−^ internal; *in vivo* recording		
**RETINA**				
*Bipolar cells*	n.s.	BIC (20)		Hull et al., 2006
	~42 pA	TPMPA (150)		
		Goldfish (*C. auratus*); –60 mV; high Cl^−^ internal		
	~22 pA	PTX (50)	ρ1, ρ2	Palmer, 2006; Jones and Palmer, 2011
	~16 pA	TPMPA (50)		
	n.s.	BIC (50)		
		Goldfish (*C. auratus*); –60 mV; high Cl^−^ internal; 18–23°C; NBQX (5)		
*Retinal ganglion cells*	−2.3 ± 3 pA, n.s.	GBZ (5); Rat (Sprague–Dawley), 1–7 day		Wang et al., 2007
	−0.5 ± 2.0 pA, n.s.	GBZ (5); Mouse (C57Bl/6), 0–7 day		
		–60 mV; high Cl^−^ internal; 32–34°C		
*Starburst amacrine cells*	48.7 ± 16 pA	GBZ (5); Mouse (IL2RA-GFP), 5–7 day	δ	Wang et al., 2007
		–60 mV; high Cl^−^ internal; 32–34°C		

*Magnitude of tonic current as measured in slice electrophysiology with the animals, recording conditions, and antagonists used. GABA_A_Rs responsible for the tonic conductance are noted*.

a *BLA, basolateral amygdala; CeA, central amygdala; CRH, corticotropin-releasing hormone; FS, fast-spiking; GnRH, gonadotropin-releasing hormone; LA, lateral amygdala; LGN, lateral geniculate nucleus; LTS, low-threshold spiking; MGB, medial geniculate body; ML, molecular layer; PVN, paraventricular nucleus; RS, regular-spiking; RTN, reticular thalamic nucleus; RVLM, rostral ventrolateral medulla; SON, supraoptic nucleus; SST, somatostatin; TC, thalamocortical; TMN, tuberomammillary nucleus; VB, ventrobasal thalamic nucleus*.

b *Parenthesis denotes percentage of cells exhibiting a tonic conductance; n.s., not significant*.

c *BIC, bicuculline; CNQX, 6-cyano-7-nitroquinoxaline-2,3-dione; CPP, 3-(2-carboxypiperazin-4-yl)propyl-1-phosphonic acid; DNQX, 6,7-dinitroquinoxaline-2,3-dione; GBZ, gabazine (SR95531); KYN, kynurenic acid; MCPG, α-methyl-4-carboxyphenylglycine; MSOP, α-methylserine-O-phosphate; NBQX, 2,3,-dihydroxy-6-nitro-7-sulfamoyl-benzo[f]quinoxaline-2,3-dione; PTX, picrotoxin; PTZ, pentylenetetrazol; THIP, 4,5,6,7-tetrahydroisoxazolo[5,4-c]pyridin-3-ol; TPMPA, (1,2,5,6-tetrahydropyridin-4-yl)methylphosphinic acid; TTX, tetrodotoxin*.

Many studies have measured the tonic current in DGGCs using different GABA_A_R antagonists, but the results have been somewhat variable (Table 1). One potential explanation for the variability in the recorded tonic currents is the pharmacology of different GABAergic antagonists used in the recordings. While picrotoxin blocks the chloride pore of GABA_A_Rs, bicuculline and gabazine are antagonists at the GABA binding site, and so are not able to prevent conductance of chloride by spontaneous channel openings. Recently, it has been suggested that the predominantly gabazine-resistant tonic current in DGGCs is maintained by spontaneous openings of GABA_A_Rs, especially at *in vivo* concentrations of GABA (<200 nM) at which DGGCs detect negligible amounts of the ambient neurotransmitter (Wlodarczyk et al., 2013). These findings suggest that at low concentrations of GABA, there is a floor effect of tonic inhibition maintained by ligand-independent, spontaneous opening of GABA_A_Rs (Walker et al., 2013). Further complicating the role of spillover in the modulation of tonic GABAergic inhibition, metabotropic GABA_B_ receptors (GABA_B_Rs) have been shown to enhance the conductance of extrasynaptic GABA_A_Rs without affecting synaptic currents, a modulation seen specifically in DGGCs (Tao et al., 2013). These GABA_B_Rs reside in the perisynaptic zone where GABA_A_R δ subunits are also found (Kulik et al., 2003). Taken together, it is possible that synaptic spillover activates extrasynaptic GABA_B_Rs, which in turn enhance the tonic GABAergic currents. Additional studies are required to clarify the role of GABA_B_Rs and the role of spillover in the modulation of tonic GABAergic inhibition.

The tonic inhibition in DGGCs, mediated by receptors containing the δ subunit, is sensitive to neurosteroid modulation (Stell et al., 2003). The binding site for neurosteroids on GABA_A_Rs has been identified on the interface between the α and β subunits (Hosie et al., 2006, 2009). However, incorporation of the δ subunit confers neurosteroid sensitivity (Belelli et al., 2002; Brown et al., 2002; Wohlfarth et al., 2002), acting to potentiate the tonic GABAergic inhibition at lower neurosteroid concentrations than that needed for the potentiation of the phasic current (Belelli et al., 2009). In addition to potentiating the effects of GABA on GABA_A_Rs, steroid hormones and neurosteroids have also been shown to alter the expression of GABA_A_R subunits. GABA_A_R subunit expression patterns change throughout the ovarian cycle. At times of the cycle when levels of progesterone and progesterone-derived neurosteroids, such as 3α,5α-THP (allopregnanolone), are increased, expression of the GABA_A_R δ subunit and tonic inhibition is increased in DGGCs, whereas levels of the γ2 subunit decrease (Maguire et al., 2005). It has been demonstrated that the ovarian cycle-associated changes in GABA_A_R subunit expression in the dentate gyrus is dependent on neurosteroid synthesis (Maguire and Mody, 2007). Changes in GABA_A_R subunit expression have also been demonstrated during pregnancy and the postpartum period. Elevations in neurosteroid levels in the brain and plasma during pregnancy (Concas et al., 1998; Follesa et al., 1998) are accompanied by a downregulation of GABA_A_R γ2 and δ subunits in the dentate gyrus, resulting in a decrease in both tonic and phasic inhibition (Maguire and Mody, 2008; Maguire et al., 2009). It has been proposed that the downregulation of GABA_A_R subunit expression during pregnancy is a compensatory mechanism to offset the massive increase in neurosteroid levels during pregnancy which can act to potentiate GABAergic inhibition (Maguire et al., 2009; Maguire and Mody, 2009). Consistent with this theory, the increased hippocampal excitability in slices from pregnant mice was restored to virgin levels upon the addition of allopregnanolone (Maguire et al., 2009; Maguire and Mody, 2009). These results suggest that homeostatic mechanisms exist to balance GABA_A_R expression with fluctuating hormone levels, which likely functions to maintain an ideal level of neuronal excitability (Maguire and Mody, 2009). Neurosteroid-mediated changes in GABA_A_R subunit expression in the dentate gyrus have also been demonstrated following acute stress. Acute stress can rapidly increase the level of stress hormones like THDOC, and acute restraint stress has been demonstrated to upregulate the GABA_A_R δ subunit and increase tonic inhibition in DGGCs (Maguire and Mody, 2007). This GABA_A_R regulation was shown to be dependent on neurosteroidogenesis (Maguire and Mody, 2007; Sarkar et al., 2011), demonstrating dynamic regulation of GABA_A_Rs in the dentate gyrus under conditions of altered neurosteroid levels (for review see Ferando and Mody 2012).

#### CA1

Pyramidal cells in the CA1 subfield are responsible for the output of the hippocampus and the activity of these neurons is regulated by both tonic and phasic GABAergic inhibition. GABA_A_Rs containing α5 subunits are expressed at a high density in this area (Sperk et al., 1997) and are responsible for mediating the majority of tonic inhibition in these neurons (Caraiscos et al., 2004) (Table 1). The tonic current mediated by these α5 GABA_A_Rs provides shunting inhibition that limits the excitability of CA1 neurons (Bonin et al., 2007).

GABA_A_Rs containing the α5 subunit are sensitive to modulation by amnestic drugs (Chambers et al., 2003). Though it was initially determined that the tonic inhibition in CA1 pyramidal cells is not sensitive to neurosteroid modulation (Stell et al., 2003), further studies have demonstrated that in the presence of low concentrations of ambient GABA, GABA_A_Rs containing the δ subunit are activated to produce a tonic current, while in the presence of higher concentrations of ambient GABA, α5-containing GABA_A_Rs contribute to the tonic current in CA1 pyramidal cells (Scimemi et al., 2005). Thus, both δ - and α5-containing receptors may contribute to the tonic current in CA1 pyramidal cells (Table 1), and the ambient concentration of GABA may determine which subtypes are activated.

#### Subiculum

Most of the output of the hippocampus from CA1 goes through the subiculum to parahippocampal networks, including other parts of the limbic system (O'Mara et al., 2001). GABAergic regulation in the subiculum keeps intrinsically bursting cells strongly inhibited (Menendez de la Prida, 2003). Both the α5 and δ subunits have been implicated in the regulation of the pyramidal cells of the subiculum (Curia et al., 2009) (Table 1). Furthermore, a more detailed analysis of specific cell types demonstrated that regular spiking cells and interneurons have similar magnitudes of tonic inhibition, while intrinsically bursting cells experience greater tonic currents (Panuccio et al., 2012) (Table 1). These data demonstrate the variability of tonic GABAergic inhibition within different cell types in the subiculum.

### Cerebellum

The cerebellum is best known for its role in motor coordination and learning, though it has also been implicated in other functions such as cognitive and affective processes (Wolf et al., 2009; Rochefort et al., 2013). The unique cytoarchitecture of the cerebellar cortex which is a highly regular structure consisting of granule cells that send parallel fibers to Purkinje cells, which in turn project to the deep nuclei that control the output of the cerebellum, reflects the information flow through this structure. Mossy fibers from outside the cerebellum bring excitatory input to granule cells, and these synapses are regulated by GABAergic inhibition from local interneurons known as Golgi cells. The Purkinje cells also receive GABAergic modulation from stellate and basket cells. The information is processed by the Purkinje cells which control the outflow from the cerebellar cortex.

Early studies found GABA_A_Rs on the cell bodies of granule cells that were not associated with synapses (Somogyi et al., 1989), and revealed a persistent GABAergic inhibition of these cells (Kaneda et al., 1995). This tonic inhibition was partly from spillover of synaptically-released GABA (TTX-sensitive), and partly from a source other than vesicular release (TTX-insensitive) (Kaneda et al., 1995). Extrasynaptic GABA_A_Rs containing α6 and δ subunits were identified on the dendrites of cerebellar granule cells (Nusser et al., 1998) and proposed to mediate the tonic GABAergic inhibition of these cells (Table 1). Interestingly, the tonic current becomes less dependent upon action potentials as the cerebellum matures (Wall and Usowicz, 1997). During development, the contribution from phasic currents decreases, while the contribution from the tonic current increases (Brickley et al., 1996). Thus, tonic GABAergic inhibition plays a critical role in regulating the excitability of cerebellar granule cells.

### Neocortex

Early electrophysiological experiments described the presence of a tonic GABA_A_R-mediated inhibition of pyramidal neurons from cortical slices (Salin and Prince, 1996). Expression of the GABA_A_R δ subunit has been identified in the cortex (Pirker et al., 2000). Consistent with the role of the GABA_A_R δ subunit in mediating tonic GABAergic inhibition in the cortex, tonic inhibition in layer 2/3 and layer 5 neurons is enhanced with 4,5,6,7-tetrahydroisothiazolo-[5,4-c]pyridine-3-ol (THIP), a superagonist acting preferentially at GABA_A_R δ subunit-containing receptors (Drasbek and Jensen, 2006) (Table 1). The tonic inhibition in neurons from layer 5 is lower in magnitude compared to layer 2/3, likely due to the lower expression of the δ subunit in layer 5 (Drasbek and Jensen, 2006). Further experiments indicated against the involvement of α1, α2, α3, or γ2 subunits in mediating the tonic conductance of layer 2/3 pyramidal cells, leaving α4β δ GABA_A_Rs as the probable candidate (Drasbek et al., 2007) (Table 1). The activity of GATs regulate the amount of GABA present in the synapse as well as the concentration detected by extrasynaptic receptors, thus modulating phasic and tonic inhibition in layer 2/3 pyramidal neurons as well as layer 1 interneurons (Keros and Hablitz, 2005). Neurogliaform cells are present in the somatosensory cortex. They possess GABA_A_Rs with a δ subunit as well as a tonic conductance, and they can provide a nonsynaptic source of extracellular GABA to surrounding neurons (Olah et al., 2009). In contrast, somatostatin-expressing interneurons do not express a tonic conductance (Vardya et al., 2008).

Interestingly, the complexity of tonic GABAergic inhibition in the cortex is highlighted by the fact that THIP paradoxically increases network excitability in layer 4 circuits (Krook-Magnuson and Huntsman, 2005). This THIP-induced increase in network excitability is likely due to the increased sensitivity of low-threshold spiking (LTS) interneurons to THIP compared to principal neurons in layer 4 of the mouse barrel cortex (Krook-Magnuson and Huntsman, 2005). Similarly, THIP is thought to preferentially dampen interneuron activity over layer 2/3 neurons, resulting in a decrease in the frequency of sIPSCs and an increase in the frequency of sEPSCs (Drasbek and Jensen, 2006). These surprising findings demonstrate that the effects of tonic inhibition and pharmacological agents cannot be understood in isolation and must be considered in the context of the larger network.

### Amygdala

The amygdala comprises several nuclei in the medial temporal lobe (Sah et al., 2003). It is involved in emotional learning, particularly in forming associations between sensory stimuli and emotional reactions. The function of the amygdala has largely been studied using fear conditioning and extinction, in which robust inhibitory circuits regulate the expression of conditioned responses, with neurons in the lateral and central nuclei exhibiting firing rates that are among the lowest in the brain (Quirk and Gehlert, 2003). Thus, inhibition appears to play a critical role in the proper functioning of the amygdala.

Neurons in the basolateral amygdala (BLA) project to the medial sector of the central amygdala (CeA) (Ehrlich et al., 2009). BLA neurons exhibit a tonic GABAergic conductance (Wu et al., 2007) which is enhanced by THIP (Olmos-Serrano et al., 2010) (Table 1). Although the use of THIP implicates the δ subunit in mediating the tonic GABAergic inhibition in neurons of the BLA, experiments with the α3-selective agonist TP003 activated a tonic current in these neurons (Marowsky et al., 2012). Furthermore, mice with deficits in the α3 subunit (*Gabra3^−/−^* mice) exhibit a loss of the tonic conductance in BLA principal neurons (Marowsky et al., 2012). Since TP003 acts at the benzodiazepine binding site, this also suggests the presence of a γ2 subunit. While all BLA principal cells display a tonic current, only 70% of principal cells in the lateral amygdala (LA) express a tonic conductance (Marowsky et al., 2012). However, the α3 subunit does not appear to be involved in mediating the tonic current in the LA (Marowsky et al., 2012). In the CeA, tonic GABAergic inhibition in CRF1 neurons is mediated by receptors containing α1 subunits, and depends on action potential-dependent release of GABA (Herman et al., 2013). In contrast, the tonic conductance in non-CRF1 neurons depends on the δ subunit, and is enhanced by ethanol (Herman et al., 2013). These data demonstrate the variability and the role of specific subunits in mediating the tonic inhibiton in the different subregions of the amygdala.

### Striatum

In the striatum, a nucleus of the basal ganglia involved in motor behavior and habit formation, over 95% of the neurons are medium spiny neurons (MSNs), which are GABAergic projection neurons that give rise to two major pathways of motor output. Approximately half of the MSNs express dopamine D1 receptors and regulate the direct pathway of movement, a circuit that initiates and executes voluntary movements. The other half expresses dopamine D2 receptors and regulates the indirect pathway, a circuit that suppresses unwanted movements. These projection neurons are in turn regulated by cholinergic and GABAergic interneurons within the striatum (Tepper et al., 2010; Goldberg and Reynolds, 2011).

In juvenile mice, D2-positive MSNs showed a greater magnitude of tonic inhibition compared to D1-positive cells (Ade et al., 2008). This tonic conductance was shown to be TTX-sensitive, indicating a synaptic origin of GABA, and is mediated by receptors with α5 and β 3 subunits (Ade et al., 2008; Janssen et al., 2009, 2011) (Table 1). The level of tonic currents in these cells are limited by the activity of GAT1 (Kirmse et al., 2008), but are independent of GAT2/3 activity (Kirmse et al., 2009), confirming the role of spillover in mediating the tonic current in the MSNs of the striatum. As the striatum matures, the MSNs undergo changes in their GABA_A_R subunit expression profile, with α5 subunits being downregulated while α4 and δ subunits are upregulated (Laurie et al., 1992b). As a result of this developmental switch, D1-positive MSNs in adult mice exhibit tonic currents mediated by the δ subunit with a greater magnitude than D2-positive neurons (Santhakumar et al., 2010) (Table 1). These data demonstrate the cell-specific diversity and developmental regulation of tonic GABAergic inhibition in the striatum.

### Thalamus

The thalamus acts as a relay station for sensory and motor information between cortical and subcortical areas. Subunit composition of GABA_A_Rs contributes to differences in GABAergic conductance between different thalamic nuclei (Table 1). While cells in the ventrobasal nuclei (VB) undergo a developmental switch postnatally in their expression of GABA_A_R subunits, neurons in the nucleus reticularis thalami (NRT) do not undergo such a switch (Laurie et al., 1992b). Thalamocortical (TC) neurons in the dorsal lateral geniculate nucleus (dLGN) and the VB express extrasynaptic GABA_A_Rs that mediate a tonic conductance (Cope et al., 2005; Jia et al., 2005). In contrast, no tonic current was observed in NRT cells, which do not express α4 or δ subunits (Cope et al., 2005; Jia et al., 2005). Mediated by α4β 2δ GABA_A_Rs, the tonic conductance in VB neurons contributes about 80% of the total GABAergic transmission, and is relevant to the actions of hypnotic compounds (Belelli et al., 2005; Chandra et al., 2006; Herd et al., 2009) (Table 1). Relay neurons in the dLGN express a tonic conductance that is mediated by GABA_A_Rs that contain a δ subunit, while neurons in the adjacent ventral LGN lack α4 or δ subunits and do not exhibit a tonic current (Bright et al., 2007). Similar to neurons in the dLGN and VB nuclei, neurons of the medial geniculate body exhibit a tonic current mediated by α4β δ GABA_A_Rs (Richardson et al., 2013) (Table 1). Thus, tonic inhibition in the thalamic nuclei is very diverse and likely contributes to the unique functions of each of these subregions.

### Hypothalamus

The hypothalamus controls several neuroendocrine systems and homeostatic mechanisms, including body temperature, food intake, thirst, circadian rhythms, and arousal. It is involved in behaviors relating to aggression, reproduction, stress, and sleep. Bidirectional neural fibers connect the hypothalamus to other parts of the brain as well as to the autonomic nervous system, while it also functions in a neurosecretory fashion. A diverse brain region, the hypothalamus is made up of eleven major nuclei as well as less distinct areas. Here we discuss the role of tonic GABAergic inhibition in a few of the prominent hypothalamic cell types (Table 1).

#### Gonadotropin-releasing hormone (GnRH) cells

Cells in the preoptic area release GnRH to regulate the release of follicle-stimulating hormone and luteinizing hormone from the anterior pituitary. GnRH neurons receive strong GABAergic input and express numerous GABA_A_R subunits (Sim et al., 2000; Sullivan et al., 2003). GnRH mRNA levels are decreased in *Gabrg2*^−/−^ mice (Simonian et al., 2000), suggesting a role for the GABA_A_R γ2 subunit in the regulation of GnRH neurons. Nevertheless, a GnRH neuron-specific conditional knockout further revealed that removal of the γ2 subunit reduced the amplitude and frequency of IPSCs, but had no effects on fertility, estrous cycles, puberty onset, or levels of luteinizing hormone (Lee et al., 2010a). Interestingly, these findings suggest that phasic GABAergic signaling does little to modulate the effectiveness of GnRH release. However, much less is known about the role of tonic GABAergic inhibition in GnRH release and reproductive health. Approximately half of the GnRH cells express mRNA for the δ subunit, and about the same proportion display a tonic current, though tonic GABAergic signaling can be induced in more GnRH neurons if either neuronal or glial GATs are blocked (Bhattarai et al., 2011) (Table 1). The tonic current is not dependent on synaptic release, and instead regulates the resting membrane potential in both juvenile and adult GnRH cells (Bhattarai et al., 2011).

Further complicating the role of GABAergic control of GnRH neurons is evidence that the actions of GABA may not always be inhibitory. During development, GnRH neurons exhibit depolarizing actions of GABA; whereas, GnRH neurons from adults appear to display both hyperpolarizing and depolarizing responses to GABA (Bhattarai et al., 2011; Herbison and Moenter, 2011). The heterogeneity in the actions of GABA on GnRH neurons and the impact on the regulation of GnRH neurons is difficult to resolve. Additional studies are required to elucidate the role of GABAergic signaling on the control of GnRH neurons and the impact on reproductive health.

#### Oxytocin and vasopressin cells

Magnocellular neurosecretory cells in the supraoptic nucleus (SON) and the paraventricular nucleus (PVN) are responsible for secreting oxytocin or vasopressin from the posterior pituitary. In the SON, these neurons have been shown to be under robust GABAergic inhibition (Wuarin and Dudek, 1993). Phasic GABAergic currents in SON cells are modulated by neurosteroids, especially surrounding parturition, and these changes are attributed to changes in GABA_A_R subunit composition (Brussaard et al., 1997; Koksma et al., 2003). In addition to IPSCs, the activity of SON cells is also restrained by a tonic GABAergic conductance that can be enhanced by blocking the activity of glial GATs (Park et al., 2006). The tonic inhibitory control of these neurons is likely mediated by benzodiazepine-sensitive α5βγ2 GABA_A_Rs and is sensitive to neurosteroid potentiation (Jo et al., 2011) (Table 1).

#### PVN-RVLM projection neurons

In the PVN, a subset of neurons projects to the rostral ventrolateral medulla (RVLM) to control sympathetic output. In these RVLM-projecting neurons, a THIP-sensitive tonic conductance was discovered to constrain the firing rate of these neurons, causing downstream effects including decreased renal sympathetic nerve activity, arterial pressure, and heart rate (Park et al., 2007, 2009) (Table 1). THIP is a preferential agonist at GABA_A_R δ subunit-containing receptors, suggesting a role of these receptors in the regulation of RVLM-projecting neurons in the PVN. However, the evidence that there is a gabazine-insensitive tonic current in these neurons (Park et al., 2007) (Table 1) suggests that spontaneous openings of GABA_A_Rs may also play a role in the regulation of these neurons. Additional evidence suggests that the tonic activity in these presympathetic PVN neurons is driven by spontaneous GABA release at the synapse as well as regulated by glial GATs (Park et al., 2009). Neuronal-glial interactions are thus involved in maintaining autonomic homeostasis through modulating ambient GABA levels.

#### Corticotropin-releasing hormone (CRH) neurons

Among the parvocellular neurosecretory cells in the PVN, CRH neurons initiate the body's physiological response to stress. These cells release CRH to stimulate the release of adrenocorticotropic hormone (ACTH) from the anterior pituitary, which leads to the secretion of cortisol from the adrenal cortex in humans and corticosterone in mice. Following chronic stress, a downregulation of the GABA_A_R δ subunit has been observed in the PVN (Verkuyl et al., 2004), implicating extrasynaptic receptors in stress reactivity. Consistent with the role of the GABA_A_R δ subunit in the regulation of CRH neurons, CRH neurons exhibit a tonic conductance which is sensitive to neurosteroid modulation (Sarkar et al., 2011) (Table 1). Furthermore, the tonic inhibition of CRH neurons and neurosteroid sensitivity is absent in mice deficient in the GABA_A_R δ subunit (*Gabrd*^−/−^ mice) (Sarkar et al., 2011). These data demonstrate a role for the GABA_A_R δ subunit in the regulation of CRH neurons. However, the role of these receptors in the regulation of CRH neurons following stress is much more complicated. Following acute stress, there are alterations in the expression of the K^+^/Cl^−^ co-transporter 2 (KCC2) in the PVN, which maintains the chloride gradient in adult neurons (Rivera et al., 1999, 2005) and is required for effective GABAergic transmission. Dephosphorylation of KCC2 residue Ser940 and downregulation of KCC2 results in a collapse of the chloride gradient and depolarizing, excitatory actions of GABA on CRH neurons (Hewitt et al., 2009; Sarkar et al., 2011). These data suggest that downregulation of KCC2 is required to overcome the robust GABAergic constraint on CRH neurons to trigger the stress response (Sarkar et al., 2011). This synaptic regulation of CRH neuron activity represents a novel control mechanism, distinct from the classical action of glucocorticoid signaling through gene transcription (Tasker and Herman, 2011; Levy and Tasker, 2012) and highlights the complex role of GABAergic regulation of CRH neurons.

### Spinal cord

In the spinal cord, cells are organized by function, and display regional variation in the signals used to transfer information (Table 1). The dorsal horn is innervated by different sensory modalities. The neurons of substantia gelatinosa (lamina II) constitute an important relay station in nociception. Signals from pain fibers are regulated by GABAergic and glycinergic inhibition from neurons in lamina II. Tonic GABAergic inhibition could be found or induced in a subset of these neurons in adult mice and rats (Ataka and Gu, 2006; Takahashi et al., 2006; Maeda et al., 2010). In contrast, juvenile rats did not display a tonic GABAergic conductance, although a tonic glycinergic conductance was observed (Mitchell et al., 2007). The tonic GABAergic current is mediated at least in part by GABA_A_R δ subunit-containing receptors, and enhancing this current with THIP can reduce nociception in behavioral assays of pain (Bonin et al., 2011). These data show that tonic inhibition plays a role in pain control, and suggest that loss of tonic inhibition may contribute to hypersensitization. In addition, inhibitory neurons in lamina I and the outer part of lamina II (IIo) mainly receive GABAergic inputs, and they display a tonic GABAergic conductance that increases as juveniles mature to adulthood (Takazawa and MacDermott, 2010). However, inhibitory neurons along the border between lamina II and III receive predominantly glycinergic inputs, and their tonic GABAergic conductance diminishes over the same course of postnatal development (Takazawa and MacDermott, 2010). This regional separation allows for specific sensory circuits to be tuned through distinct mechanisms.

In the intermediolateral (IML) cell column of the lateral horn, sympathetic preganglionic neurons direct sympathetic outflow from the CNS, and are regulated by local interneurons. Although application of GABA_A_R antagonists elicited no change under resting conditions, tonic inhibition could be induced in sympathetic preganglion neurons if the clamped cell was depolarized to 0 mV, or if the chloride driving force was modified by changing the intracellular solution of the patch pipette (Wang et al., 2008). These effects were diazepam-sensitive but zolpidem-insensitive, suggesting that under certain conditions, these neurons exhibit the capacity for tonic inhibition mediated by α5βγ2 GABA_A_Rs, consistent with findings of α5 expression but not δ in this region (Wang et al., 2008). In contrast, no evidence of tonic inhibition was seen in local interneurons from the same region (Wang et al., 2008). A tonic conductance has also been observed in ventral horn neurons of chick embryos, and was estimated to arise from approximately 30 GABA_A_Rs located extrasynaptically, compared to about 10 synaptic GABA_A_Rs mediating a phasic current (Chub and O'Donovan, 2001). The afferents and efferents of the spinal cord traverse the boundary between the central and peripheral nervous systems. Other studies have investigated the role of GABA_A_Rs in spinal roots and peripheral ganglia, but the primary focus of this review is tonic GABAergic inhibition in the CNS.

### Retina

As an outgrowth of the developing diencephalon, the retina can be considered part of the CNS. In the retina, inhibitory chloride conductances are mediated by typical GABA_A_Rs as well as atypical GABA_A_Rs that contain ρ subunits. During perinatal development, tonic inhibition can be induced in retinal ganglion cells and starburst amacrine cells, blocking spontaneous waves of activity that sweep through the retina (Wang et al., 2007). The tonic conductance in starburst amacrine cells are THIP-sensitive, consistent with the presence of the GABA_A_R δ subunit on their processes (Wang et al., 2007). A tonic current also regulates membrane excitability in the terminals of bipolar cells (Hull et al., 2006; Palmer, 2006), which pass information from the photoreceptors to the ganglion cells that make up the optic nerve. This tonic conductance is not dependent on vesicular release or transporter reversal, and is thought to be mediated by heteromeric ρ 1-ρ 2 receptors and homomeric ρ 1 receptors (Jones and Palmer, 2009, 2011). These findings highlight the diversity of GABA_A_Rs and the brain region and cell-type specificity of tonic GABAergic inhibition (Table 1).

## Tonic and phasic inhibition in interneurons

**Figure 1:**
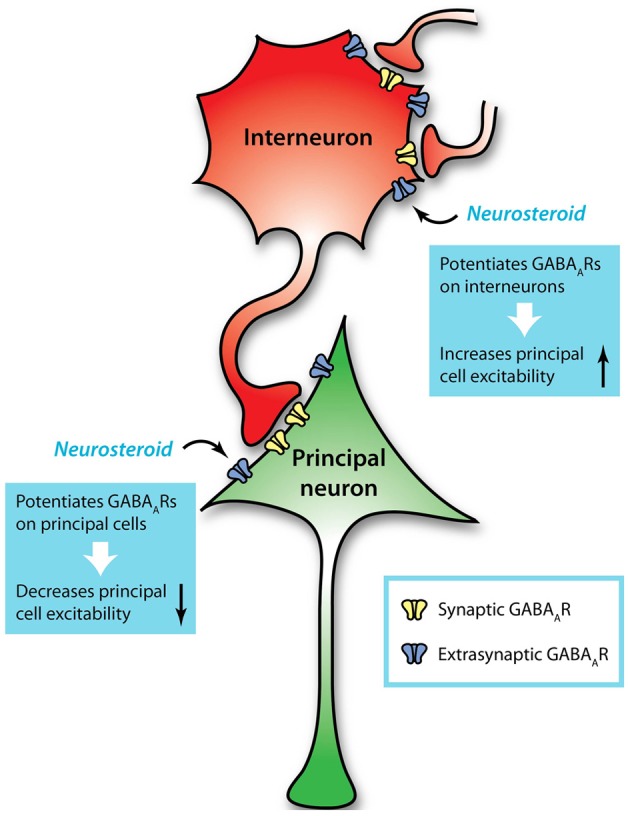
**Extrasynaptic GABA_A_Rs mediate tonic conductances in principal neurons and interneurons**. Interneurons regulate the activity of principal cells through GABAergic transmission, but are in turn regulated by GABAergic input from other interneurons. This additional level of GABAergic signaling introduces complexity to understanding neuronal excitability and its modulation. For example, neurosteroids can act on GABA_A_Rs, especially extrasynaptic receptors that contain a δ subunit, to potentiate GABAergic inhibition on principal neurons and diminish their excitability. Simultaneously, neurosteroids can potentiate GABAergic inhibition on interneurons, thus disinhibiting the principal neurons and enhancing their excitability. These opposing effects, combined with the variability in GABAergic inhibition across cell types and brain regions, present complications when considering GABA_A_Rs as therapeutic targets.

The majority of studies investigating tonic inhibition have focused on its effectiveness in limiting the excitability of principal neurons. However, interneurons in several brain regions also express extrasynaptic GABA_A_Rs and have been shown to be regulated by tonic GABAergic inhibition. In the dentate gyrus, interneurons in the molecular layer display a tonic current that is mediated by α1βδ GABA_A_Rs (Glykys et al., 2007; Lee and Maguire, 2013) (Table 1). Similarly, basket cells in the dentate gyrus display a shunting conductance that plays an important role in the ability of interneurons to generate network oscillations (Vida et al., 2006). In the CA1 subregion of the hippocampus, both stratum radiatum and stratum oriens interneurons show tonic currents that inhibit their excitability, limiting their inhibitory signaling to pyramidal cells (Semyanov et al., 2003) (Table 1). The tonic inhibition in stratum radiatum interneurons is mediated, at least in part, by δ subunit-containing GABA_A_Rs (Lee and Maguire, 2013). Further studies demonstrate that the tonic conductance of hippocampal interneurons exhibits a biphasic effect depending on extracellular GABA concentrations (Song et al., 2011) (Table 1). At low concentrations of extracellular GABA, the activity of extrasynaptic GABA_A_Rs will produce an excitatory depolarization on these neurons. As the GABA concentration increases, the tonic conductance becomes shunting and then hyperpolarizing (Song et al., 2011). In addition, interneurons of CA3 have low levels of KCC2 throughout development (Banke and McBain, 2006), leading to *E*_*GABA*_ values that are higher than the resting membrane potential, such that GABAergic transmission would depolarize the cell membrane. These data demonstrate a complex role for GABAergic regulation of interneurons in the hippocampus (Figure 1) and suggest a crucial role for tonic inhibition.

While much attention has been given to the role of interneurons in regulating the activity of principal neurons, this essential function itself requires strict regulation (Chamberland and Topolnik, 2012). In imagining the impact of interneurons on principal neurons, the effect on a principal neuron seems somewhat straightforward (Figure 1). However, when considering the larger neuronal network, in which interneurons communicate with one another as well as with numerous principal neurons, the computational complexity increases dramatically (Figure 1), but allows for dynamic systems that display rhythmic, oscillatory activity. Different subtypes of interneurons can serve distinct functions in controlling network excitability by targeting different compartments of the principal neuron, as well as targeting each other (Lovett-Barron et al., 2012). Studies investigating the impact of GABAergic inhibition on network function have largely focused on the role of synaptic inhibition. Thus, the role of tonic conductances in interneurons is still not fully understood within the healthy neuronal network. However, studies have implicated tonic GABAergic inhibition of interneurons in playing a critical role in the generation of gamma oscillations (Mann and Mody, 2010), suggesting an important role for neuronal network activity.

## Function of tonic inhibition

Traditionally, the neuron is depicted as performing summations of excitatory and inhibitory signals from its synaptic inputs and, if the sum exceeds a threshold, generating an all-or-none output response in the form of firing an action potential. Further study has yielded a better appreciation of the range and sophistication of computational mechanisms across different types of neurons. These various lines of evidence point to special roles of tonic signaling complementary to the functions of synaptic transmission. This section will focus on the role of tonic GABAergic inhibition in regulating neuronal excitability and in the generation of rhythmic activity and neuronal oscillations.

### Tonic inhibition and neuronal excitability

Extrasynaptic GABA_A_Rs generate a persistent hyperpolarizing current which makes it less likely to generate an action potential. Even in the case of depolarizing GABAergic currents, the tonic conductance decreases the membrane resistance, making the neuron less sensitive to sharp changes in voltage, thus attenuating the effect of excitatory input at the synapse. This shunting inhibition not only offsets the input-output relationship of cerebellar granule cells, but also changes its gain (slope) (Mitchell and Silver, 2003). It has also been demonstrated that tonically active GABA_A_Rs in CA1 pyramidal cells exhibit outward rectification, indicating that the magnitude of the tonic current will depend on the voltage at which it is measured (Pavlov et al., 2009). At subthreshold membrane potentials, the tonic conductance will have little impact on neuronal firing, but the conductance will be larger at firing threshold potentials. Thus, in these cells, the extrasynaptic GABA_A_Rs mediate a tonic current that modulates neuronal offset, not gain (Pavlov et al., 2009). The specific cell type being investigated might explain these differing conclusions, as cerebellar granule cells and CA1 pyramidal cells may employ different mechanisms to encode information (Silver, 2010).

### Generation of rhythmic activity and network oscillations

Gamma oscillations have been observed over a variety of cognitive states, including memory processing, exploratory behavior, and consciousness (Bragin et al., 1995; Lisman and Idiart, 1995; Llinas et al., 1998). This network activity is thought to underlie a neural coding scheme used to represent information in the environment (for review see Lisman and Buzsaki 2008). GABAergic inhibition has been shown to play a critical role in the generation of rhythmic activity and network oscillations (for review see Bartos et al. 2007). Furthermore, the connectivity between interneurons and principal neurons is essential for the generation of this oscillatory activity.

Interneurons of the hippocampus demonstrate variety in the input sources and targets of their axons (Freund and Buzsaki, 1996). Some exhibit extensive arborizations that allow individual interneurons to communicate with hundreds of principal neurons. Through these connections, an interneuron can use rhythmic inhibition to phase-lock the intrinsic oscillatory patterns of pyramidal cells and synchronize their firing through post-inhibitory rebound activation (Cobb et al., 1995). Synchronized spiking represent a means of encoding information that is independent of the firing rate, and can be recognized by coincidence detectors downstream (Mann and Paulsen, 2007; Ainsworth et al., 2012). Interneurons that make perisomatic synapses onto principal neurons have been implicated in the generation of network oscillations (Mann et al., 2005). The identity of these interneurons is likely fast-spiking, parvalbumin-positive basket cells (Bartos et al., 2007; Lapray et al., 2012). Gap junction coupling between interneurons helps to ensure robust synchrony in their output and increase the range of the oscillations (Fukuda and Kosaka, 2000; Tamas et al., 2000; Traub et al., 2003). In addition, parvalbumin-expressing axo-axonic cells have firing patterns that differ from basket cells and offer a distinct contribution to oscillation dynamics (Massi et al., 2012). Interneurons may also receive recurrent, excitatory feedback from the principal neurons, or may be inherently capable of generating oscillatory activity independently (Mann and Paulsen, 2007). The complex connectivity on the cellular and subcellular level likely drives the generation of neuronal oscillations.

The majority of studies of network oscillations have focused on the role of synaptic GABAergic inhibition. However, elegant studies have also implicated tonic GABAergic inhibition of interneurons in limiting the frequency of gamma oscillations (Mann and Mody, 2010). Consistent with this hypothesis, mice with deficits of specific GABA_A_R subunits exhibit abnormal oscillatory patterns (Huntsman et al., 1999; Nusser et al., 2001; Towers et al., 2004; Lagier et al., 2007; Mann and Mody, 2010). This section clearly highlights the importance of interneurons in the generation of network oscillations and more recent studies suggest that tonic GABAergic inhibition may also play a critical role in generating this type of activity. Information regarding the function of tonic GABAergic inhibition and the involvement of specific subtypes of GABA_A_Rs can also be gleaned from studies investigating the consequences of the loss of specific GABA_A_R subunits resulting from genetic manipulations in animal models and mutations in human studies.

## Human mutations in extrasynaptic GABA_A_Rs

Human studies investigating mutations in GABA_A_Rs associated with numerous diseases have primarily focused on genes encoding for synaptic GABA_A_Rs. Yet studies investigating mutations in extrasynaptic GABA_A_R subunits can provide clues into the function of these receptor subtypes. This section will review the limited number of studies investigating human mutations identified in the genes encoding for extrasynaptic GABA_A_Rs and known associations for epilepsies.

### GABRD

Missense point mutations within the human *GABRD* gene, which encodes for the GABA_A_R δ subunit, have been associated with generalized epilepsies with febrile seizures plus (GEFS+). Studies in heterologous systems have demonstrated that these two mutations on the extracellular domain of the δ subunit, E177A and R220H, create GABA_A_Rs with diminished currents due to a shorter duration of open time (Dibbens et al., 2004; Feng et al., 2006). In addition, a R220H mutation has been identified in the *GABRD* gene in the general population with unknown association (Dibbens et al., 2004). These data suggest that the GABA_A_R δ subunit impacts neuronal excitability and deficits in the functioning of these receptors have been implicated in the development of epilepsy.

### GABRA5

Few studies have investigated potential mutations in the *GABRA5* gene, encoding for the GABA_A_R α5 subunit. A single study conducted a mutation analysis of 50 patients with childhood absence epilepsy (CAE) but did not find any associations with the gene that encodes the α5 subunit (Feucht et al., 1999). Clearly, further studies are required to determine if there are mutations in the *GABRA5* gene associated with specific diseases.

### GABRA6

A single mutation in the *GABRA6* gene (R46W) has been identified and associated with CAE (Dibbens et al., 2009). Studies in heterologous systems demonstrate that the R46W mutation in the gene encoding for the α6 subunit affects the gating and assembly of α6β 2δ and α6β 2γ2L receptors, reducing their open times, burst durations, and current density (Hernandez et al., 2011). Similar to mutations in the *GABRD* gene, mutations in the *GABRA6* may contribute to the development of epilepsy.

### GABRB3

Three mutations in the *GABRB3* gene, encoding for the β 3 subunit, have been identified, P11S, S15F, and G32R, which are associated with CAE (Tanaka et al., 2008). Further, the P11S variant has also been associated with autism, in which seizures are often comorbid (Tanaka et al., 2008; Delahanty et al., 2011). The P11S and S15F mutations are located in the signal peptide region, while the G32R mutation is located at the N-terminal end of the protein. All three mutations result in GABA_A_Rs that are hyperglycosylated and have reduced GABAergic currents (Tanaka et al., 2008; Gurba et al., 2012). These studies demonstrate that mutations in *GABRB3* impair GABAergic inhibition mediated by these receptors and may contribute to the development of epilepsy.

Together these studies demonstrate that human mutations identified in genes encoding for extrasynaptic GABA_A_Rs are associated with epilepsy syndromes (for review see Macdonald et al. 2010). Studies in mouse models have enabled a closer look at the impact of these receptors on neuronal excitability and their role in epilepsy, which will be summarized in the following section.

## Knockout mice and excitability

Studies in mouse models have facilitated investigation into the function of specific extrasynaptic GABA_A_R subunits. The section below details studies investigating the knockout of genes encoding for extrasynaptic GABA_A_R subunits, focusing on the impact on neuronal excitability, seizure susceptibility, and epileptogenesis.

### *Gabrd* knockout

*Gabrd* knockout mice (*Gabrd*^−/−^ mice) have provided a useful tool for studying the role of GABA_A_R δ subunit-containing receptors in regulating neuronal excitability. *Gabrd*^−/−^ mice exhibited a loss of tonic inhibition in DGGCs and cerebellar granule cells which was essential for demonstrating the importance of these receptors in mediating the tonic inhibition (Brickley et al., 2001; Stell et al., 2003). Tonic inhibition mediated by GABA_A_R δ subunit-containing receptors has been suggested to dampen excitability and even maintain the “dentate gate” (Coulter and Carlson, 2007), preventing excessive excitation. Despite this proposed role of the δ subunit in regulating neuronal excitability, surprisingly, there are no reports of spontaneous seizures in *Gabrd*^−/−^ mice. However, it is important to note that *Gabrd*^−/−^ mice do exhibit an increased sensitivity to convulsive seizures induced by pentylenetetrazol (PTZ) (Spigelman et al., 2002), demonstrating the importance of this subunit in dampening and/or regulating neuronal excitability.

*Gabrd*^−/−^ mice have also been useful in clarifying the role of the δ subunit in conferring neurosteroid sensitivity to GABA_A_Rs. Mice deficient in the GABA_A_R δ subunit exhibit reduced sensitivity to neuroactive steroids, indicating that GABA_A_Rs containing a δ subunit contribute to the actions of neurosteroids on GABA_A_Rs (Mihalek et al., 1999). The effects of neurosteroids on GABAergic inhibition are reduced in numerous cell types from *Gabrd^−/−^* mice, including cerebellar granule cells, thalamic relay neurons in the VB, and DGGCs (Vicini et al., 2002; Porcello et al., 2003; Spigelman et al., 2003). These data support a role for the GABA_A_R δ subunit in conferring neurosteroid sensitivity despite the fact that the neurosteroid binding site has been identified on the α/β interface (Hosie et al., 2006, 2009).

### *Gabra5* knockout

Though the δ subunit mediates tonic inhibition in many brain regions, extrasynaptic GABA_A_Rs containing the α5 subunit play a role in mediating tonic inhibition in specific brain regions, such as in principal neurons in the CA1 and CA3 subfields of the hippocampus. Mice deficient in the GABA_A_R α5 subunit (*Gabra5*^−/−^ mice) exhibit a reduced tonic inhibitory conductance in CA1 and CA3 pyramidal neurons, resulting in network hyperexcitability in the form of spontaneous epileptiform activity in field recordings (Caraiscos et al., 2004; Glykys and Mody, 2006). However, given the proposed role of this subunit in dampening excitability, again it is surprising that no spontaneous seizures were observed in these mice. The loss of α5 subunits in CA3 led to a deficit in the ability to modulate the power of gamma oscillations in proportion to changes in the overall network activity (Towers et al., 2004). These data demonstrate a role for the GABA_A_R α5 subunit in mediating the tonic GABAergic inhibition in principal neurons in specific hippocampal subregions.

### *Gabra6* knockout

The α6 knockout mice (*Gabra6*^−/−^ mice) provided a useful tool for demonstrating the association of α6 and δ subunits in extrasynaptic receptors of cerebellar granule cells (Jones et al., 1997; Nusser et al., 1999). The loss of these subunits in *Gabra6*^−/−^ mice abolished tonic GABAergic inhibition in the cerebellar neurons, yet their excitability was unchanged (Brickley et al., 2001). Closer studies revealed that the lack of effect on neuronal excitability in mice lacking the GABA_A_R α6 subunit was due to a compensatory increase in leak conductance by a two-pore-domain K+ channel (TASK-1) (Brickley et al., 2001). These findings highlight the importance of the GABA_A_R α6 subunit in mediating tonic GABAergic inhibition in cerebellar granule cells and also point to compensatory mechanisms following the loss of tonic inhibition which will be discussed in a later section.

### *Gabrb3* knockout

Mice with deficits in the *Gabrb3* gene (*Gabrb3*^−/−^ mice) display a complicated phenotype (Homanics et al., 1997), including seizures. Loss of the β 3 subunit results in altered sleep (Wisor et al., 2002), likely due to the high expression of the β 3 subunit in the reticular nucleus of the thalamus (NRT), which consists of inhibitory neurons that drive sleep-related oscillations through recurrent collaterals. *Gabrb3*^−/−^ mice demonstrate a dramatic reduction of sIPSCs in these neurons, and this loss of inhibition leads to hypersynchronous oscillations, a condition that has been observed in absence epilepsy (Huntsman et al., 1999). The mice also have reduced mIPSCs in the inhibitory granule cells of the olfactory bulb, and stronger mIPSCS in the principal neurons, mitral/tufted cells (Nusser et al., 2001). Theta and gamma frequency oscillations in the network were enhanced, and the mice demonstrated changes in the ability to distinguish between various alcohols. However, the impact of loss of the β 3 subunit on tonic GABAergic inhibition and the impact on neuronal excitability remain less clear.

### Compensatory mechanisms in global knockout models

From the phenotypes of global knockout mice, it appears that mice lacking extrasynaptic GABA_A_Rs have less dramatic changes in excitability compared to mice with loss of synaptic receptors. However, this may not necessarily mean that the impact of specific subunits is more or less important. Care must be taken when interpreting results from global knockout mice because of the possibility of compensatory effects by other genes. For example, the tonic conductance in molecular layer interneurons is mediated by both GABA_A_Rs containing a δ subunit and those that contain an α5 subunit. Yet only the double knockout mouse shows a lack of tonic inhibition; disrupting the *Gabrd* gene or the *Gabra5* gene individually does not diminish tonic currents in these interneurons (Glykys et al., 2008). These compensatory changes highlight the importance of maintaining GABAergic inhibition. In *Gabrd*^−/−^ mice, there is a residual tonic current mediated by the α5 subunit; likewise, in *Gabra5*^−/−^ mice, there is a residual conductance mediated by the δ subunit (Glykys et al., 2008). However, compensatory effects are not always predictable. While α6 knockout mice also lack the δ subunit in their cerebellar granule cells (Jones et al., 1997; Nusser et al., 1999), the converse is not also true, as δ knockouts do not lack α6 subunits (Tretter et al., 2001). Instead, there is an upregulation of γ2 subunit expression in the cerebellum, suggesting that γ2 replaces δ in co-assembling with α6 subunits (Tretter et al., 2001; Korpi et al., 2002). Similarly in the forebrain, where δ subunits appear to preferentially assemble with α4 subunits, *Gabrd* knockout mice exhibit decreased expression of α4, while γ2 is upregulated and substitutes for δ in joining with the remaining α4 subunits (Korpi et al., 2002; Peng et al., 2002). In α4 knockout mice, DGGCs do not show compensatory upregulation of α6 subunits to partner with δ, and thus the expression of δ is greatly reduced in DGGCs (but unaffected in interneurons) (Glykys et al., 2007). These findings highlight the complexity of GABA_A_R compensation which must be considered when using knockout mice.

Neither are compensatory mechanisms limited to other GABA_A_R subunits. Loss of the α6 and δ subunits from cerebellar granule cells in *Gabra*6^−/−^ mice results in the compensatory upregulation of TASK-1 (Brickley et al., 2001). Not only can the loss of GABA_A_Rs be compensated by other kinds of channels, GABA_A_Rs can also be recruited to compensate for the loss of other types of conductances. Hyperpolarization-activated cyclic nucleotide-gated (HCN) channels mediate the *I*_*h*_ current that serves to stabilize the membrane potential against fluctuations. Knockout mice that lack the HCN1 isoform were expected to exhibit enhanced network activation and seizure phenotypes, but the GABA_A_R α5 subunit was shown to be upregulated and compensated for the loss of HCN1 by providing an increased tonic conductance (Chen et al., 2010). Such plasticity demonstrates the flexibility of neurons in maintaining proper inhibitory regulation to prevent hyperexcitability.

These compensatory changes can potentially confound the interpretation of studies investigating the significance of individual GABA_A_R subunits, the function of synaptic versus extrasynaptic receptors, and the roles of phasic and tonic conductances. Cre-lox recombination offers a more selective targeting of individual genes which can potentially avoid some of the compensatory mechanisms seen in global knockout mice. Mice with a floxed gene offer the flexibility of conditionally removing that gene from specific target cells that express Cre recombinase. To date, few studies have taken advantage of these systems to study the role of individual GABA_A_R subunits in specific brain regions and cell types.

### Conditional *Gabrb3* knockout

Since the global *Gabrb3* knockout strain showed levels of neonatal mortality and compensation that made it difficult to determine the role of the β 3 subunit, a floxed β 3 mouse was generated to facilitate the creation of conditional knockout lines (Ferguson et al., 2007). A recombination-mediated global knockout strain recapitulated the phenotype observed with the traditional knockout line (Homanics et al., 1997). Forebrain-selective knockout of the β 3 subunit resulted in spontaneous seizures, suggesting a role for GABA_A_R β 3-containing receptors in regulating neuronal excitability and the development of epilepsy. The floxed β 3 mice have also been used to generate a conditional knockout strain that selectively removes the β 3 subunit from D2-positive MSNs in the striatum, resulting in a loss of tonic GABAergic inhibition in these neurons (Janssen et al., 2011). These data demonstrate a role for the GABA_A_R β 3 subunit in mediating tonic inhibition and constraining neuronal excitability.

### Conditional *Gabrd* knockout

Recently, our laboratory has generated floxed *Gabrd* mice to facilitate investigation into the role of GABA_A_R δ subunit-mediated tonic inhibition and its impact on neuronal excitability in specific cell types and brain regions. The floxed *Gabrd* mice have been used to remove the GABA_A_R δ subunit from GAD65-positive interneurons (*Gabrd*/*Gad* mice). Loss of the GABA_A_R δ subunit from GAD65-positive interneurons resulted in a loss of tonic GABAergic inhibition in interneurons in the stratum radiatum and the molecular layer of the dentate gyrus (Lee and Maguire, 2013), which has been shown to be mediated, at least in part, by δ subunit-containing receptors (Glykys et al., 2007, 2008). Furthermore, loss of the GABA_A_R δ subunit in GAD65-positive interneurons increased both the tonic and phasic inhibition onto DGGCs and CA1 pyramidal neurons (Lee and Maguire, 2013). The increased inhibition onto principal neurons in *Gabrd*/*Gad* mice is associated with decreased excitability of both DGGCs and CA1 pyramidal neurons as well as decreased susceptibility to kainic acid-induced seizures (Lee and Maguire, 2013).

Recent studies were also conducted in our laboratory to investigate the role of the GABA_A_R δ subunit in the regulation of CRH neurons. Loss of the GABA_A_R δ subunit specifically in CRH neurons (*Gabrd*/*Crh* mice) results in a loss of tonic GABAergic inhibition of these neurons (Lee et al., 2013). Furthermore, the neurosteroid modulation of the tonic current is lost in *Gabrd^−/−^* mice (Sarkar et al., 2011), demonstrating that the GABA_A_R δ subunit confers neurosteroid sensitivity to CRH neurons. The loss of tonic GABAergic inhibition in CRH neurons from *Gabrd*/*Crh* mice is associated with an increased firing rate of these neurons (Lee et al., 2013). The generation of *Gabrd*/*Crh* mice was essential for demonstrating the role of the GABA_A_R δ subunit in the control and neurosteroid sensitivity of CRH neurons, suggesting a novel mechanism for HPA axis regulation.

Further studies are required to investigate the role of extrasynaptic GABA_A_R subunits in different cell types and brain regions. However, evidence from global and conditional GABA_A_R subunit knockout mice suggest a role for extrasynaptic GABA_A_Rs in regulating neuronal excitability and in epilepsy. Thus, the following section will review the role of extrasynaptic GABA_A_R subunits in pathological conditions, focusing particularly on the hippocampus in the context of temporal lobe epilepsy.

## Experimental models of epilepsy

It is widely accepted that systemic administration of kainic acid (KA) or pilocarpine in rats can recapitulate the clinical and neuropathological aspects of temporal lobe epilepsy (TLE) (Ben-Ari et al., 1980; Turski et al., 1983). The following section will review the literature describing alterations in tonic GABAergic inhibition and the expression of extrasynaptic GABA_A_Rs in these experimental models of TLE and comparison to post-mortem observations in patients with TLE.

### Changes in extrasynaptic receptors and tonic inhibition

Numerous changes in GABA_A_Rs have been documented in both experimental models of epilepsy as well as in patients with epilepsy (for review see Sperk et al. 2009). Changes in the distribution of various GABA_A_R subunits have been documented in the hippocampus following seizures induced with the chemoconvulsant kainic acid. Following acute seizures induced with kainic acid, there are alterations in the expression of numerous GABA_A_R subunits, including upregulation of the α1and β2 subunits and a reduction in the expression of the α2, α5, and δ subunits (Schwarzer et al., 1997; Tsunashima et al., 1997). It is interesting to note the reduction in the expression of the GABA_A_R subunits mediating tonic GABAergic inhibition, such as the α5 and δ subunits, following acute kainic acid-induced seizures. In the chronic epileptic state following kainic acid-induced status epilepticus, there is an upregulation of the α1, α2, α4, β 2, β 3, and γ2 subunits (Schwarzer et al., 1997; Tsunashima et al., 1997). Interestingly, both the extrasynaptic δ and α5 subunits are downregulated in the chronic epileptic state (Schwarzer et al., 1997; Tsunashima et al., 1997). Changes in GABA_A_R subunit expression have also been observed in the subiculum, entorhinal cortex, and perirhinal cortex in a kainic acid model of epileptogenesis (Drexel et al., 2013). These parahippocampal regions display an initial reduction of all subunits following acute seizures (Drexel et al., 2013). Later, by the chronic phase, α1, α2, α3, and γ2 subunits recovered and even became upregulated in some areas; in contrast, α5, β 1, β 2, β 3, and δ subunits remained chronically downregulated (Drexel et al., 2013). These data suggest that alterations in GABA_A_R subunit expression, in particular the GABA_A_R δ subunit, may play a role in epilepsy. However, it remains unclear whether these changes are part of the pathological process or compensatory mechanisms.

Many changes in GABA_A_R subunit expression have also been documented in the pilocarpine model of TLE. At the mRNA level, an increase in the α4, β 3, δ, and ε subunits have been observed in DGGCs (Brooks-Kayal et al., 1998); whereas, a decreased expression of the α1 and β 1 was observed in DGGCs from epileptic rats (Brooks-Kayal et al., 1998). A closer look at changes in protein expression of the GABA_A_R δ subunit in the dentate gyrus of pilocarpine-treated mice demonstrate a progressive loss of δ subunit labeling throughout the molecular layer of the dentate gyrus (Peng et al., 2004). Accordingly, neurosteroid enhancement of tonic inhibition was lost from the DGGCs in pilocarpine-treated mice, consistent with the loss of neurosteroid-sensitive extrasynaptic GABA_A_Rs that contain a δ subunit (Peng et al., 2004; Zhang et al., 2007). Interestingly, there is an upregulation of the GABA_A_R δ subunit in molecular layer interneurons following pilocarpine-induced epilepsy (Peng et al., 2004). It has been proposed that the downregulation of the extrasynaptic GABA_A_R δ subunit-containing receptors mediating tonic GABAergic inhibition in DGGCs and upregulation in molecular layer interneurons may increase excitability and contribute to the epileptogenic process. However, the magnitude of the tonic current was unchanged in DGGCs in the chronic period, as the loss of α4β δ GABA_A_Rs was compensated by replacement with α4β γ2 receptors, shown by a net shift of γ2 subunit expression from synaptic to extrasynaptic locations (Peng et al., 2004; Zhang et al., 2007). These results are comparable to changes observed in epileptic rats after electrically-induced status epilepticus (Nishimura et al., 2005; Rajasekaran et al., 2010). In contrast, others have noted a long-lasting enhancement of tonic currents in the chronic phase mediated by GABA_A_Rs with an α5 subunit, in addition to existing α4β δ GABA_A_Rs (Zhan and Nadler, 2009). Interpretation of the changes in GABA_A_R subunit expression is difficult as they may contribute to either the epileptogenic process or compensatory changes to counteract the epileptogenic process.

### Comparison to human tissue

While these experimental models have been able to replicate many facets of temporal lobe epilepsy, including important characteristics of seizure activity, disease progression, and neuropathology, studies of human tissue are necessary to validate the experimental findings from animal models. However, there are caveats to evaluating the results obtained from postmortem tissue since tissue integrity may be compromised to variable degrees between subjects. Despite these caveats, studies using postmortem tissue from patients with TLE have identified alterations in GABA_A_R subunit expression (Loup et al., 2000; Pirker et al., 2003). A decreased expression of the GABA_A_R α1 has been observed in patients with TLE; whereas, an increased expression of the α2, α3, α4, α5, β 2, β 3, γ2, and δ subunits have been observed in patients with TLE (for review see Sperk et al. 2009). Consistent with the increased expression of the majority of GABA_A_R subunit expression, studies using resected hippocampal tissue from patients with epilepsy suggest that GABAergic inhibition is preserved or even enhanced (Babb et al., 1989; Mathern et al., 1995; Wittner et al., 2001). Thus, it remains unclear if or how alterations in GABA_A_R subunit expression contribute to epilepsy. One of the difficulties in interpreting changes in GABA_A_R subunit expression is understanding in which cell types these changes are occurring and the impact on the larger neuronal network.

## Conclusions

A number of GABA_A_R isoforms are capable of mediating tonic GABAergic inhibition, depending on the specific brain region and cell type. There is heterogeneity in the magnitude of GABAergic inhibition recorded across various cell types (Table 1), which can change dramatically based on differences in GABA_A_R subunit expression, extracellular GABA concentration, presence or absence of modulators, and changes in GABAergic uptake mechanisms. To understand the impact of tonic GABAergic inhibition on network activity, we must consider the brain regions, cell types, and GABA_A_R subtypes involved. For instance, the fact that both principal neurons and inhibitory neurons exhibit tonic conductances, often mediated by similar GABA_A_R subtypes, would produce potentially offsetting net effects for modulation (Figure 1), which complicates targeting these receptors for therapy. Further studies are required to understand the complex role of tonic GABAergic inhibition on network function under both physiological and pathological conditions.

### Conflict of interest statement

The authors declare that the research was conducted in the absence of any commercial or financial relationships that could be construed as a potential conflict of interest.
